# An atlas on risk factors for type 2 diabetes: a wide-angled Mendelian randomisation study

**DOI:** 10.1007/s00125-020-05253-x

**Published:** 2020-09-08

**Authors:** Shuai Yuan, Susanna C. Larsson

**Affiliations:** 1 Unit of Cardiovascular and Nutritional Epidemiology, Institute of Environmental Medicine, Karolinska Institutet, Nobelsväg 13, 17177 Stockholm, Sweden; 2 Department of Surgical Sciences, Uppsala University, Dag Hammarskjölds Väg 14B, 75185 Uppsala, Sweden

**Keywords:** Mendelian randomisation, Prevention, Review, Risk factors, Type 2 diabetes

## Abstract

**Aims/hypothesis:**

The aim of this study was to use Mendelian randomisation (MR) to identify the causal risk factors for type 2 diabetes.

**Methods:**

We first conducted a review of meta-analyses and review articles to pinpoint possible risk factors for type 2 diabetes. Around 170 possible risk factors were identified of which 97 risk factors with available genetic instrumental variables were included in MR analyses. To reveal more risk factors that were not included in our MR analyses, we conducted a review of published MR studies of type 2 diabetes. For our MR analyses, we used summary-level data from the DIAbetes Genetics Replication And Meta-analysis consortium (74,124 type 2 diabetes cases and 824,006 controls of European ancestry). Potential causal associations were replicated using the FinnGen consortium (11,006 type 2 diabetes cases and 82,655 controls of European ancestry). The inverse-variance weighted method was used as the main analysis. Multivariable MR analysis was used to assess whether the observed associations with type 2 diabetes were mediated by BMI. We used the Benjamini–Hochberg method that controls false discovery rate for multiple testing.

**Results:**

We found evidence of causal associations between 34 exposures (19 risk factors and 15 protective factors) and type 2 diabetes. Insomnia was identified as a novel risk factor (OR 1.17 [95% CI 1.11, 1.23]). The other 18 risk factors were depression, systolic BP, smoking initiation, lifetime smoking, coffee (caffeine) consumption, plasma isoleucine, valine and leucine, liver alanine aminotransferase, childhood and adulthood BMI, body fat percentage, visceral fat mass, resting heart rate, and four plasma fatty acids. The 15 exposures associated with a decreased risk of type 2 diabetes were plasma alanine, HDL- and total cholesterol, age at menarche, testosterone levels, sex hormone binding globulin levels (adjusted for BMI), birthweight, adulthood height, lean body mass (for women), four plasma fatty acids, circulating 25-hydroxyvitamin D and education years. Eight associations remained after adjustment for adulthood BMI. We additionally identified 21 suggestive risk factors (*p* < 0.05), such as alcohol consumption, breakfast skipping, daytime napping, short sleep, urinary sodium, and certain amino acids and inflammatory factors.

**Conclusions/interpretation:**

The present study verified several previously reported risk factors and identified novel potential risk factors for type 2 diabetes. Prevention strategies for type 2 diabetes should be considered from multiple perspectives on obesity, mental health, sleep quality, education level, birthweight and smoking.

**Graphical abstract:**

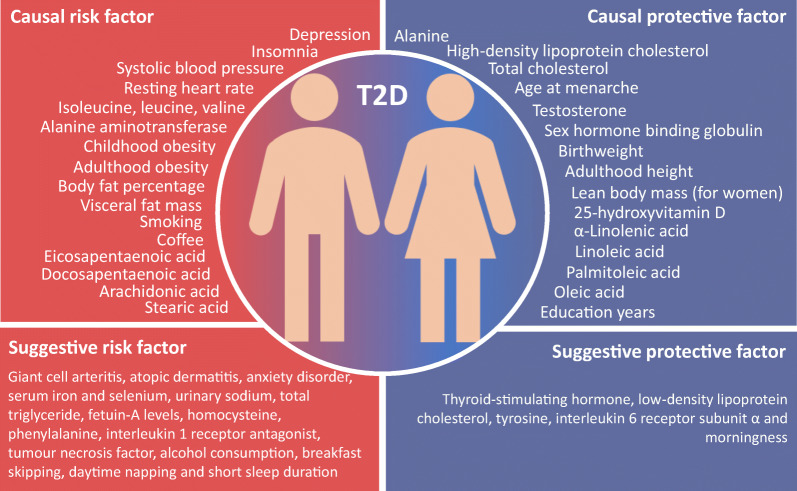

**Electronic supplementary material:**

The online version of this article (10.1007/s00125-020-05253-x) contains supplementary material, which is available to authorised users.

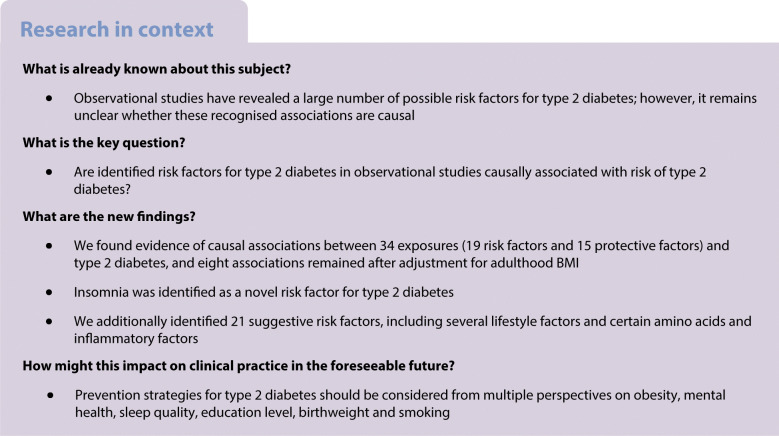



## Introduction

Type 2 diabetes is a global public health issue, affecting 9 in 100 adults worldwide in 2015 according to the International Diabetes Federation [1, 2]. The increasing prevalence of type 2 diabetes along with severe complications cause an immense disease and economic burden [1, 3]. Therefore, it is important to better understand the aetiological basis of type 2 diabetes and establish prevention strategies.

Reviews of observational studies have revealed a large number of possible risk factors for type 2 diabetes covering health status, dietary and lifestyle factors, environmental factors, and different biomarkers [4–6]. However, whether the reported associations are causal remains unclear due to potential methodological limitations in the observational studies, such as confounding and reverse causality. For certain exposures with defined detrimental influences on human health, such as smoking and heavy alcohol drinking, it is unfeasible to determine their causal associations with type 2 diabetes in an experimental setting.

**Fig. 1:**
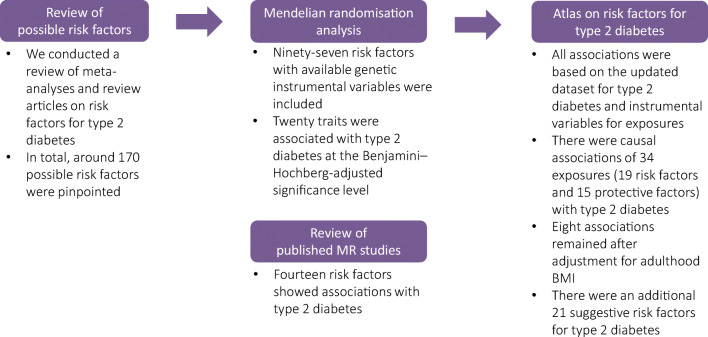
Overview of study design

Utilising genetic variants as instrumental variables for an exposure, Mendelian randomisation (MR) analysis can strengthen the causal inference on an exposure–outcome association [7, 8]. The MR study design has two major strengths when compared with the traditional observational design (electronic supplementary material [ESM] Fig. 1). First, the result of an MR analysis is less likely to be driven by confounding because genetic variants are randomly allocated at conception and, therefore, one trait is generally unrelated to other traits. This resembles the random assignment of participants to experimental and control groups in an RCT [7–9]. Second, an MR analysis avoids reverse causality as alleles are fixed and cannot be modified by the onset and progression of a disease [7, 8].

Several previous MR studies have examined the associations of various exposures, such as nutritional, lifestyle, obesity-related, hormone-related and inflammatory factors and internal biomarkers, with type 2 diabetes (ESM Table 1). However, there has been no study systematically assessing the causal effects of possible risk factors on type 2 diabetes. Here, we conducted an MR investigation to determine the causal associations of a wide range of possible risk factors proposed by observational studies with type 2 diabetes risk.

## Methods

### Study design overview

The overview of study design is presented in Fig. 1. To identify possible risk factors for type 2 diabetes, we conducted a review of meta-analyses and review articles identified by a search in the PubMed database. In total, around 170 possible risk factors were pinpointed of which 97 risk factors with available genetic instrumental variables were included in the present MR study. In addition, we conducted a review of published MR studies of type 2 diabetes to reveal more risk factors that were not included in the present study. This MR study was approved by the Swedish Ethical Review Authority.

### Data source for type 2 diabetes

Summary-level data for type 2 diabetes were available in the DIAbetes Genetics Replication And Meta-analysis (DIAGRAM) consortium, which includes 32 studies with a total of 898,130 individuals of European descent (74,124 cases and 824,006 controls) [10]. Participants had a mean age of around 55 years and 51.8% were men. The Haplotype Reference Consortium (HRC) reference panel was used in the imputation stage and adjustments were made for population structure (e.g. through principal components), relatedness and study-specific covariates. Considering that BMI may mediate the associations between certain exposures and type 2 diabetes, we used the summary-level estimates from the genome-wide association analysis without BMI adjustment.

Data from the FinnGen consortium (11,006 type 2 diabetes cases and 82,655 controls) were used in the replication stage (https://www.finngen.fi/fi). Detailed methods (e.g. participating biobanks/cohorts, data collection, genotyping and data analysis) are presented in its webpage.

### Data source for adulthood BMI

Summary-level data for BMI were obtained from a recent genome-wide association study (GWAS) of 806,834 individuals of European ancestry (including Genetic Investigation of ANthropometric Traits [GIANT] consortium and UK Biobank) [11]. The HRC reference panel was used in the imputation stage and adjustments were made for age, sex and principal components of ancestry. Results were validated in an independent dataset including 7721 UK individuals.

### Instrumental variable selection

SNPs associated with each exposure at the genome-wide significance threshold (*p* < 5 × 10^−8^) were proposed as instrumental variables for 97 exposures from corresponding GWASs. We excluded SNPs in linkage disequilibrium (*R*^2^ ≥ 0.01). For each trait, all instrumental variables were harmonised so that the effect alleles reflected the allele associated with increased probability, prevalence or levels of the exposure. For SNPs not available in the type 2 diabetes dataset, proxy SNPs were searched in the dataset of the National Cancer Institute, Division of Cancer Epidemiology & Genetics (https://ldlink.nci.nih.gov/) by setting *R*^2^ ≥ 0.8. We replaced missing SNPs with proxy SNPs for traits with ≤10 missing SNPs. Detailed information for data sources and SNPs used is shown in ESM Table 2. Definitions for exposures are presented in ESM Table 3.

### A review of previous MR studies

Many associations were tested in previous studies with an MR design. In order to reveal more causal risk factors for type 2 diabetes, we conducted a review of previous MR studies of possible risk factors for type 2 diabetes. Detailed methods are shown in the ESM Methods. In total, 238 papers were screened, and 40 individual studies were included. Publication data, number of SNPs used as instrumental variables, outcome source information and effect estimates (95% CIs) were extracted.

### Statistical analysis

The *F*-statistic was estimated to examine the strength of the genetic instrument for each exposure and an *F*-statistic above 10 was considered a sufficiently strong instrument. The inverse-variance weighted method was used as the main analysis [12]. For exposures instrumented by ≥3 SNPs, the overall estimates were calculated using the random-effects inverse-variance weighted method. For exposures with only 1 or 2 SNPs available as instrumental variables, the fixed-effects inverse-variance weighted method was used. The inverse-variance weighted method can provide the most precise estimates but is sensitive to invalid instrumental variables and pleiotropic effects [12]. We additionally performed several sensitivity analyses based on the weighted median [13] and MR-Egger regression [14] methods to test the consistency of the associations under the different assumptions and to detect possible pleiotropy. Assuming that more than 50% of the weight in the analysis comes from valid instrumental variables, the weighted median method can provide an accurate estimate of the causal effect of the exposure on outcome [13]. MR-Egger regression can identify and correct for directional pleiotropy, but the estimation is of low precision [14]. We used *I*^2^ to represent the heterogeneity among used SNPs [15] and defined the horizontal pleiotropy according to the *p* value for the intercept in the MR-Egger model [16]. Considering the partial overlap of participants included in the GWASs of certain exposures and the DIAGRAM consortium, we replicated the associations with *p* < 0.05 in the inverse-variance weighted model using an independent GWAS dataset—FinnGen consortium. To evaluate whether the exposures that were associated with type 2 diabetes at *p* ≤ 0.05 in the univariable analysis have a direct effect on type 2 diabetes not mediated by BMI, we performed a multivariable random-effects inverse-variance weighted analysis [17] adjusting for adulthood BMI. Since we aimed at assessing the effect of exposure of interest only, we adopted the multivariable MR method testing for mediation by BMI, rather than allowing for an independent effect of BMI as well as mediation by BMI simultaneously.

ORs and 95% CIs of type 2 diabetes were scaled to the unit reported in ESM Table 2. We conducted a general power analysis using a webtool [18] for MR analysis (ESM Table 4). All analyses were performed in Stata/SE 15.0 using the mrrobust [19] and in R 3.6.0 using the MendelianRandomization package [20]. All estimates were reported with two-tailed *p* values. We used the Benjamini–Hochberg method that controls the false discovery rate (FDR) for multiple testing [21]. Results from this analysis are presented in ESM Table 5. Associations with a Benjamini–Hochberg adjusted *p* value <0.05 were regarded as significant. Suggestive causal associations were defined based on a comprehensive consideration of *p* values for the estimates in inverse-variance weighted method and/or weighted median method and the consistency across analyses (*p* < 0.05 in inverse-variance weighted or weighted median models and the direction of the association remained consistent in two models).

## Results

### Summary of results of this MR investigation and review of previous MR studies

**Fig. 2:**
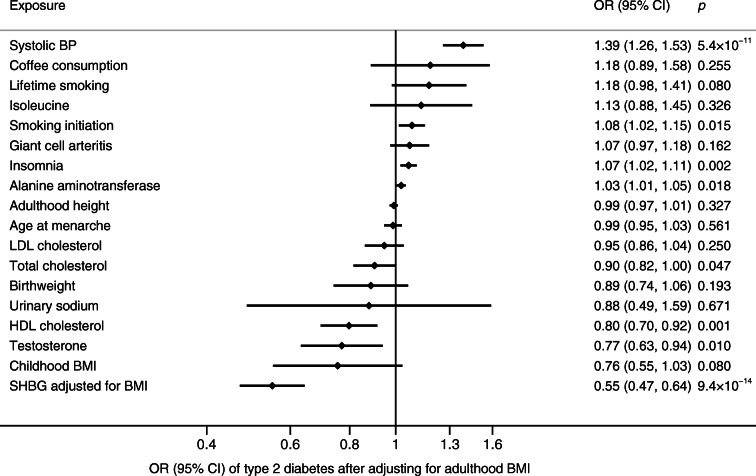
Associations of risk factors identified for type 2 diabetes in the univariable MR analysis with type 2 diabetes after adjustment for adulthood BMI. Traits that highly correlated with adulthood BMI, such as visceral fat mass and body fat percentage, were not included. SHBG, sex hormone binding globulin

Among 97 exposures examined in this MR investigation (Table 1 and ESM Table 6), 29 were nominally associated with type 2 diabetes (*p* < 0.05) of which 20 were associated with type 2 diabetes after Benjamini–Hochberg adjustment for multiple comparisons (ESM Tables 5 and 6). Combining those 20 significant associations with 14 significant associations (Table 2) identified by our review of previous MR studies (ESM Table 1), we found evidence of causal associations of 34 exposures with risk of type 2 diabetes. In detail, an increased risk of type 2 diabetes was observed with 19 exposures: depression, insomnia, systolic BP, smoking initiation, lifetime smoking, coffee (caffeine) consumption, plasma isoleucine, valine and leucine, liver alanine aminotransferase, childhood and adulthood BMI, body fat percentage, visceral fat mass, resting heart rate, and four plasma fatty acids. A decreased risk of type 2 diabetes was observed with 15 exposures: plasma alanine, HDL- and total cholesterol, age at menarche, testosterone levels, sex hormone binding globulin (SHBG) levels (adjusted for BMI), birthweight, adulthood height, lean body mass (for women), four plasma fatty acids, circulating 25-hydroxyvitamin D and education years. Eight of the associations remained in the multivariable MR analysis adjusted for adulthood BMI (Fig. 2). There were a further 21 suggestive causal factors for type 2 diabetes, including alcohol consumption, breakfast skipping, daytime napping, short sleep, urinary sodium, and certain amino acids and inflammatory factors. Details of the univariable and multivariable MR analyses and the review of previous MR studies are presented below.

**Table 1:** Associations between risk factors and type 2 diabetes in the present study using the latest DIAGRAM consortium (74,124 cases and 824,006 controls) and comparison with previous MR studies

	Previous MR study conducted (yes/no)	Published association with diabetes (if available) OR (95% CI)	PubMed identifier of published MR	Association with diabetes in the present MR study OR (95% CI)
Somatic health status				
Asthma	No	NA		1.01 (0.94, 1.10)
Atopic dermatitis	No	NA		1.05 (0.98, 1.11)
Dupuytren’s disease	No	NA		1.01 (0.99, 1.03)
Giant cell arteritis	No	NA		1.07 (1.00, 1.14)
Hyperthyroidism	No	NA		1.00 (0.95, 1.04)
Hypothyroidism	No	NA		0.99 (0.95, 1.04)
Microalbuminuria	No	NA		1.26 (0.86, 1.85)
Microvascular dysfunction	No	NA		1.03 (0.96, 1.10)
Osteoarthritis	No	NA		0.94 (0.83, 1.07)
Periodontitis	No	NA		1.03 (1.00, 1.06)
Polycystic ovary syndrome	No	NA		0.96 (0.92, 1.01)
Rheumatoid arthritis	No	NA		0.98 (0.92, 1.04)
Systolic BP	Yes	1.02 (1.01, 1.03)	27702834 [51]	1.39 (1.26, 1.54)
Telomere length	Yes	1.00 (0.84, 1.20)	28241208 [52]	1.02 (0.96, 1.08)
Mental health status				
Anorexia nervosa	No	NA		0.92 (0.84, 1.02)
Lifetime anxiety disorder	No	NA		1.05 (0.94, 1.18)
Post-traumatic stress disorder	No	NA		0.96 (0.87, 1.05)
Schizophrenia	No	NA		1.00 (0.97, 1.04)
Nutritional factor and internal biomarker				
β-carotenoid (precursor to vitamin A)	Yes	0.98 (0.91, 1.04)	19662379 [53]	0.96 (0.89, 1.05)
Retinol (vitamin A)	No	NA		1.15 (0.85, 1.56)
Vitamin B_6_	No	NA		1.00 (0.99, 1.00)
Folate (vitamin B_9_)	No	NA		0.88 (0.78, 0.99)
Vitamin B_12_	Yes	0.96 (0.71, 1.30)	29982347 [54]	0.99 (0.95, 1.04)
Vitamin C	No	NA		1.00 (0.99, 1.01)
Vitamin E	No	NA		1.21 (0.76, 1.93)
Copper	No	NA		1.03 (0.98, 1.07)
Iron	Yes	0.89 (0.81, 0.98)	30759836 [55]	1.06 (0.99, 1.13)
Magnesium	Yes	1.55 (0.26, 9.25)	30759836 [55]	1.08 (0.92, 1.26)
Potassium (urinary)	No	NA		0.71 (0.32, 1.61)
Sodium (urinary)	No	NA		2.69 (1.14, 6.34)
Selenium	Yes	1.18 (0.97, 1.43)	29788239 [56]	1.05 (1.01, 1.10)
Zinc	Yes	1.01 (0.92, 1.12)	30759836 [55]	1.00 (0.96, 1.04)
Thyroid-stimulating hormone	Yes	0.91 (0.78, 1.07)	28323940 [57]	0.96 (0.87, 1.06)
Free thyroxine	Yes	1.10 (0.94, 1.30)	28323940 [57]	0.94 (0.88, 1.01)
HDL-cholesterol	Yes	0.83 (0.76, 0.90)	27487401 [58]	0.78 (0.67, 0.91)
LDL-cholesterol	Yes	0.86 (0.80, 0.93)	27487401 [58]	0.91 (0.83, 1.00)
Total cholesterol	No	NA		0.87 (0.79, 0.96)
Total triacylglycerol	Yes	1.01 (0.91, 1.11)	27487401 [58]	1.04 (0.83, 1.29)
Lipoprotein(a)	No	NA		1.02 (0.99, 1.03)
Alanine aminotransferase	Yes	1.45 (1.10, 1.92)	31088856 [59]	1.02 (1.00, 1.03)
Alkaline phosphatase	Yes	0.91 (0.86, 0.97)	31088856 [59]	1.00 (0.98, 1.01)
γ-glutamyl transferase	Yes	0.92 (0.80, 1.06)	31088856 [59]	1.00 (0.99, 1.01)
Serum uric acid	Yes	0.95 (0.86, 1.05)	26821629 [60]	1.01 (0.87, 1.18)
Serum ferritin	Yes	0.79	26446360 [61]	1.06 (0.83, 1.36)
Fetuin-A levels	Yes	1.02 (0.97, 1.07)	29523632 [62]	1.02 (1.00, 1.05)
Bilirubin levels	Yes	0.58 (0.39, 0.84)	25368098 [63]	1.10 (0.87, 1.38)
Homocysteine	Yes	1.09 (0.92, 1.30) and 1.29 (1.09, 1.51)	26664883 [24] and 24320691 [25]	1.09 (0.95, 1.25)
Isoleucine	Yes	1.44 (1.26, 1.65)	27898682 [64]	1.26 (1.16, 1.37)
Leucine	Yes	1.85 (1.41, 2.42)	27898682 [64]	1.28 (1.10, 1.49)
Valine	Yes	1.54 (1.28, 1.84)	27898682 [64]	1.23 (1.08, 1.39)
Alanine	No	NA		0.51 (0.45, 0.58)
Phenylalanine	No	NA		1.15 (1.03, 1.29)
Tyrosine	No	NA		0.86 (0.76, 0.98)
Haemoglobin	No	NA		0.98 (0.82, 1.17)
Inflammatory factor				
TNF	No	NA		1.31 (0.90, 1.91)
C-reactive protein	Yes	1.15 (0.93, 1.42) and 1.11 (1.06, 1.17)	29753585 [22] and 30619477 [23]	1.02 (0.86, 1.20)
IgE	No	NA		1.04 (0.98, 1.11)
IL-1 receptor antagonist	Yes	0.99 (0.97, 1.10)	25726324 [65]	1.13 (1.01, 1.27)
IL-2 receptor subunit α	No	NA		0.98 (0.94, 1.01)
IL-6 receptor subunit α	Yes (IL-6 receptor)	0.97 (0.94, 1.00)	22421340 [66]	0.99 (0.98, 1.00)
IL-16	No	NA		0.97 (0.94, 1.00)
IL-17	No	NA		1.05 (0.93, 1.18)
IL-18	Yes	1.14 (1.03, 1.26)	31024619 [67]	1.00 (0.96, 1.04)
Mean platelet volume	No	NA		0.99 (0.96, 1.01)
Platelet count	No	NA		0.98 (0.92, 1.04)
Platelet distribution width	No	NA		1.01 (0.97, 1.06)
Plateletcrit	No	NA		0.96 (0.90, 1.03)
Lifestyle and sleep-related factor				
Alcohol consumption	No	NA		1.08 (0.80, 1.45)
Coffee consumption	Yes	1.02 (0.76, 1.36)	27845333 [68]	1.59 (1.09, 2.32)
Caffeine intake	No	NA		1.17 (1.09, 1.25)
Breakfast skipping	No	NA		1.72 (0.85, 3.46)
Lifetime smoking	Yes (smoking initiation)	1.28 (1.20, 1.37)	31852999 [26]	1.61 (1.36, 1.91)
Daytime napping	No	NA		1.77 (0.73, 4.24)
Sleep duration	Yes	0.85 (0.64, 1.13)	30508554 [69]	0.83 (0.62, 1.12)
Short sleep (<7 h)	No	NA		1.14 (0.92, 1.41)
Long sleep (>9 h)	No	NA		0.79 (0.47, 1.34)
Apnoea–hypopnoea index	No	NA		1.05 (0.99, 1.12)
Insomnia	No	NA		1.17 (1.11, 1.23)
Morningness	No	NA		1.00 (0.94, 1.05)
Restless leg syndrome	No	NA		1.01 (0.98, 1.05)
Moderate to vigorous physical activity	No	NA		0.69 (0.22, 2.14)
Strenuous sports or other exercises	No	NA		0.77 (0.42, 1.72)
Vigorous physical activity	No	NA		0.90 (0.78, 1.04)
Accelerometry	No	NA		0.98 (0.95, 1.02)
Sex-related factor				
Age at menarche	Yes	0.83 (0.78, 0.88)	31614369 [70]	0.84 (0.80, 0.88)
Age at menopause	No	NA		0.99 (0.96, 1.01)
Testosterone levels	Yes	1.07 (0.80, 1.43)	medRχiv [71]	0.72 (0.59, 0.89)
SHBG	Yes	0.83 (0.76, 0.91)	26050255 [72]	0.55 (0.47, 0.64)
Oestradiol levels	No	NA		0.99 (0.87, 1.13)
Obesity-related factor				
Birthweight	No	2.79 (1.90, 4.20)	31539074 [73]	0.79 (0.67, 0.92)
Childhood BMI	No	1.83 (1.46, 2.30)	29483184 [74]	1.87 (1.44, 2.44)
Adulthood BMI	No	1.31 (1.11, 1.53)	31821322 [75]	1.89 (1.73, 2.07)
Adulthood height	No	NA		0.95 (0.92, 0.98)
Body fat percentage	No	NA		2.07 (1.79, 2.39)
Visceral fat mass	No	2.50 (1.98, 3.14) for men 7.34 (4.48, 12.0) for women	31501611 [76]	2.63 (2.14, 3.23)
Circulating adiponectin	No	NA		0.82 (0.63, 1.07)
Leptin levels	No	NA		1.13 (0.24, 5.37)

NA, not available

**Table 2:** Established associations of 14 risk factors with type 2 diabetes in a review of previous MR studies using the latest DIAGRAM consortium (74,124 cases and 824,006 controls)

Risk factor	PMID	Year	SNPs	OR	95% CI	Unit
Resting heart rate	31648709 [77]	2019	Genetic score	1.12	1.11, 1.12	10 beats/min
α-Linolenic acid	31690987 [78]	2019	1	0.93	0.90, 0.96	SD
Eicosapentaenoic acid	31690987 [78]	2019	2	1.08	1.03, 1.12	SD
Docosapentaenoic acid	31690987 [78]	2019	2	1.04	1.02, 1.07	SD
Linoleic acid	31690987 [78]	2019	3	0.96	0.94, 0.98	SD
Arachidonic acid	31690987 [78]	2019	2	1.03	1.02, 1.05	SD
Palmitoleic acid	31690987 [78]	2019	4	0.86	0.81, 0.91	SD
Oleic acid	31690987 [78]	2019	1	0.87	0.81, 0.93	SD
Stearic acid	31690987 [78]	2019	3	1.09	1.09, 1.15	SD
25-hydroxyvitamin D	31548248 [79]	2019	7	0.94	0.88, 0.99	SD
Smoking initiation	31852999 [26]	2019	377	1.28	1.20, 1.37	NA
Fat free mass (women)	30798333 [80]	2019	311	0.91	0.84, 0.99	SD
Depression ^a^	32270255 [48]	2020	89	1.26	1.10, 1.43	Prevalence
Education years ^a^	medRχiv [49]	2020	1263	0.53	0.50, 0.57	SD

^a^Results for depression and education years were not from review and from our previous studies. The study on depression has been published, however the study on education years has not yet been officially published. The doi for education paper: 10.1101/2020.02.01.20020008

NA, not available

### Univariable MR analysis

The associations of the 97 exposures examined in this MR study with type 2 diabetes are presented in ESM Table 6. After Benjamini–Hochberg adjustment, we found evidence of causal associations of 20 exposures with type 2 diabetes. Among these exposures, 11 were associated with an increased risk of type 2 diabetes: systolic BP, lifetime smoking, insomnia, plasma isoleucine, valine and leucine, liver alanine aminotransferase, childhood and adulthood BMI, body fat percentage and visceral fat mass. Eight exposures were inversely associated with type 2 diabetes: plasma alanine, HDL- and total cholesterol, age at menarche, testosterone levels, SHBG levels (adjusted for BMI), birthweight and adulthood height. The associations for HDL-cholesterol and height with type 2 diabetes were inconsistent in the weighted median analysis. There was possible pleiotropy in the association of testosterone and SHBG levels (adjusted for BMI) with type 2 diabetes. In addition, we found suggestive evidence of associations between 21 exposures and type 2 diabetes: giant cell arteritis, atopic dermatitis, lifetime anxiety disorder, serum iron and selenium, urinary sodium, thyroid-stimulating hormone, LDL-cholesterol, total triacylglycerol, fetuin-A levels, homocysteine, phenylalanine, tyrosine, IL-1 receptor antagonist, IL-6 receptor subunit α, TNF, alcohol consumption, breakfast skipping, daytime napping, short sleep duration and morningness.

For alcohol consumption, the result of the weighted median analysis showed a positive association between alcohol consumption and type 2 diabetes (OR 1.46 [95% CI 1.16, 1.83]) (ESM Table 6). After adjustment for pleiotropy in the MR-Egger regression analysis, the OR of type 2 diabetes was 2.27 (95% CI 1.30, 3.93) per SD increase of log-transformed drinks/week (ESM Table 6). In addition, rs1229984 in the *ADH1B* gene, which is robustly associated with alcohol consumption and explains the majority of the variance of alcohol consumption, was positively associated with type 2 diabetes (OR 1.57 [95% CI 1.20, 2.07]) (ESM Fig. 2). Coffee consumption, mainly driven by SNPs in the *CYP1A1/2* and *AHR* genes which are also associated with higher caffeine intake but lower blood caffeine metabolites, was positively associated with type 2 diabetes in the inverse-variance weighted and weighted median models (ESM Table 6).

Replication results in FinnGen for associations with *p* < 0.05 in the inverse-variance model are presented in ESM Table 7. More than half of the associations reached the *p* value <0.1 and all associations were in the same direction as in the analyses based on data from the DIAGRAM consortium.

### Multivariable MR analysis

Results of the multivariable MR analysis are shown in Fig. 2. Eight risk factors remained associated with type 2 diabetes at *p* < 0.05 after adjustment for adulthood BMI. Specifically, systolic BP, smoking, insomnia and alanine aminotransferase levels were positively associated with type 2 diabetes, whereas testosterone, SHBG, and HDL- and total cholesterol levels were inversely associated with type 2 diabetes after BMI adjustment.

## Discussion

In the present MR investigation and complementary review of previous MR studies, we found evidence of causal associations of 34 exposures with type 2 diabetes. Most of the associations were identified in previous MR studies and replicated in the present MR study using a larger dataset for type 2 diabetes and/or more instrumental variables for the exposures. Insomnia was identified as a novel causal risk factor for type 2 diabetes. Eight risk factors were related to type 2 diabetes after adjustment for BMI, suggesting that those exposures affect the risk of type 2 diabetes independently of BMI. In addition, we identified 21 suggestive risk factors for type 2 diabetes, such as alcohol consumption, breakfast skipping, daytime napping, short sleep, and certain amino acids and inflammatory factors.

### Comparison with previous MR studies

Previous MR studies based on data from the largest GWAS meta-analysis of type 2 diabetes from the DIAGRAM consortium revealed associations of genetically predicted resting heart rate, plasma phospholipid levels of eight fatty acids, serum 25-hydroxyvitamin D levels, smoking initiation, lean body mass (for women), depression and education levels with type 2 diabetes. In addition, there were associations for isoleucine, leucine, valine, phylloquinone (vitamin K_1_), IL-6 receptor, IL-18, HDL- and LDL-cholesterol, alanine aminotransferase, aspartate aminotransferase, alkaline phosphatase, bilirubin levels, age at menarche, SHBG, visceral fat mass, birthweight, childhood and adulthood BMI, WHR adjusted for BMI and IGF-binding protein-3 levels with type 2 diabetes. Results for serum homocysteine and C-reactive protein levels were inconsistent.

The present MR investigation confirmed most results of previous MR studies. However, some associations did not persist when using updated data (more instrumental variables) for the exposure or outcome (more cases and controls), including associations for phylloquinone (vitamin K_1_), IL-18, liver aspartate aminotransferase, alkaline phosphatase and bilirubin. Findings were inconsistent regarding the effects of C-reactive protein [22, 23] and plasma homocysteine levels [24, 25] on type 2 diabetes in previous studies. In the present MR study, there was limited evidence supporting causal associations of C-reactive protein. However, plasma homocysteine levels showed suggestive association with type 2 diabetes. Smoking initiation has also been identified as a strong risk factor for type 2 diabetes [26]. Here, we used an instrument that predicts lifetime smoking exposure and verified the causal detrimental effect of smoking on type 2 diabetes risk.

### Novel potential risk factors

Insomnia with objective short sleep duration has been associated with an increased risk of type 2 diabetes in observational studies [27, 28]. The present MR study found strong and suggestive evidence of a causal association of insomnia and short sleep duration, respectively, with increased risk of type 2 diabetes. We did not detect an effect of sleep duration on type 2 diabetes in MR analysis assuming a linear trend. This finding is consistent with those of observational studies which have shown a U-shaped relationship between sleep duration and diabetes risk [29], although an association with long sleep duration might reflect reverse causality. In addition, observational studies have associated daytime napping with an increased risk of type 2 diabetes [30], which is supported by our MR findings. Short sleep and poor sleep quality have been shown to be associated with less healthy eating and irregular meal patterns, including breakfast skipping [31], which was related to an increased risk of type 2 diabetes in this MR study and in previous observational studies [32].

Data on the association between giant cell arteritis and type 2 diabetes are scarce. A Danish cohort study found that patients with giant cell arteritis (*n* = 1682) had a markedly increased risk of new-onset diabetes compared with the general population [33], which is in line with our finding. Even though the CIs became broader after adjustment for BMI, the OR estimate was unchanged in our study. Moreover, the opposite effects of BMI on giant cell arteritis [34] and type 2 diabetes [35] in observational studies indicates that giant cell arteritis may be a causal risk factor for diabetes independently of BMI status.

The present study provided evidence that alcohol consumption may be a risk factor for type 2 diabetes. In particular, the alcohol-raising allele of the *ADH1B* variant was strongly associated with an increased risk of type 2 diabetes. Some observational studies have indicated that light or moderate alcohol consumption is associated with a decreased risk of type 2 diabetes [36]. However, in an updated meta-analysis with 1,902,605 participants (including 125,926 individuals with type 2 diabetes), the inverse association between moderate drinking and type 2 diabetes was confined to certain subgroups [36]. Furthermore, the observed inverse association may be overestimated due to the inclusion of less healthy former drinkers in the reference group [37].

We found suggestive evidence that genetically predicted higher coffee and caffeine intake is associated with increased risk of type 2 diabetes, though the association with coffee consumption did not persist after adjustment for BMI. At first glance, these results appear contradictory to those of observational studies, which have consistently shown an inverse association between coffee consumption and type 2 diabetes incidence [38]. Nevertheless, the alleles of the variants in the *CYP1A1/A2* and *AHR* genes that predict higher caffeine consumption are related to faster caffeine metabolism and significantly lower blood levels of caffeine and higher paraxanthine-to-caffeine ratio [39]. It can thus be speculated that higher circulating levels of caffeine may be protective against type 2 diabetes.

Urinary sodium levels reflect dietary sodium to some extent. Observational studies have found that both higher urinary sodium excretion and dietary sodium intake were associated with higher risk of type 2 diabetes [40, 41], which is supported by our MR findings. In addition, consistent with the protective effects of high educational attainment on type 2 diabetes in observational studies [42], the present study confirmed that genetically predicted higher education level was associated with a lower diabetes risk. After adjustment for BMI, the association attenuated but persisted, which demonstrates that education may influence type 2 diabetes risk through BMI as well as other pathways, such as lowering psychological risk and smoking rate and levels.

Atopic dermatitis, anxiety, fetuin-A levels, phenylalanine, daytime napping and morningness were identified as novel possible causal risk factors. However, observational findings were inconclusive on atopic dermatitis [43] and anxiety [44, 45] and scarce on morningness. The established roles of fetuin-A levels [46], phenylalanine [47] and daytime napping [30] in the present study were in line with most of the observational studies. Considering limited SNPs and small variance explained by SNPs used for these traits, associations for these exposures with type 2 diabetes should be interpreted with caution and need further verification in studies in causal nature.

### BMI and other risk factors

In this study, seven of 15 exposure–type 2 diabetes associations attenuated but remained significant after adjustment for adulthood BMI, along with BMI-independent effects observed for depression [48] and education [49], which implies that adiposity is a strong risk factor for type 2 diabetes but also that controlling for BMI cannot fully prevent type 2 diabetes. Considering the large effect of obesity on type 2 diabetes [35] and an increasing global burden of obesity [50], an emphasis on weight control via healthier food choices and physical activity is needed. Simultaneously, other strategies focusing on other risk factors also merit attention, such as improving mental health status and sleep quality in developed areas, improving educational level and birthweight in developing areas and advocating anti-smoking actions worldwide.

### Strengths and limitations

This is the first study that has comprehensively assessed the causal associations between a large number of exposures and type 2 diabetes using the latest summary-level data for type 2 diabetes. The use of the MR design strengthened the causal inference on the exposure–diabetes associations due to diminished residual confounding and reverse causality. We also conducted a review to identify the main possible risk factors for type 2 diabetes. Most causal associations were replicated in an independent consortium. In addition, several sensitivity analyses were performed to test the consistency of results and reveal and correct for possible pleiotropy. Multivariable MR analysis uncovered several obesity-independent risk factors for type 2 diabetes, which provides new thinking for type 2 diabetes prevention. We used a combined design of original MR analysis and review and, therefore, extended the study scope to some extent in both risk factor detection and association revelation. Even though we pinpointed a large number of possible risk factors by conducting a review of meta-analyses and review articles on risk factors for type 2 diabetes, some risk factors may have been missed due to a scoped review design and the lack of genetic instruments for certain exposures. Another limitation is that we might have overlooked weak associations, especially for traits with small variance explained by SNPs used. However, the power would be ≥70% if the SNPs explained 1% variance of a phenotype for a risk factor with an OR ≥1.1 or ≤0.9. Another limitation is that the instrumental strength may have been low in some of the multivariable MR analyses. Instrumental variables selection was based on a mixed population for some traits, which might introduce population bias. However, the majority of participants in these corresponding GWASs were of European descent and their analyses adjusted for population principal components. Additionally, many exposures for type 2 diabetes identified in observational data cannot be assessed in the MR design due to no available instrumental variables until now.

### Conclusions

The present MR study verified several previously established risk factors and identified novel potential risk factors for type 2 diabetes using the latest summary-level data. Findings should inform public health policies for the primary prevention of type 2 diabetes. Prevention strategies should be constructed from multiple perspectives, such as lowering obesity and smoking rates and levels, and improving mental health, sleep quality, educational level and birthweight.

## Electronic supplementary material

ESM (PDF 730 kb)

## Data Availability

Genetic instruments can be obtained from the individual referenced papers. Summary-level genetic data for type 2 diabetes can be downloaded at the website: https://www.diagram-consortium.org/. The datasets analysed in this study are publicly available summary statistics.

## Abbreviations

DIAGRAM: DIAbetes Genetics Replication And Meta-analysis consortium
GWAS: Genome-wide association study
MR: Mendelian randomisation
HRC: Haplotype Reference Consortium
SHBG: Sex hormone binding globulin
