# Supplementary material for: An atlas on risk factors for type 2 diabetes: a wide-angled Mendelian randomisation study
Source: Diabetologia. 2020 Sep 8;63(11):2359–71. doi: 10.1007/s00125-020-05253-x (PMC7527357; doi:10.1007/s00125-020-05253-x)
Supplement: Supplementary file 1 — (PDF 730 kb) [file 125_2020_5253_MOESM1_ESM.pdf]

Electronic Supplementary Materials

An atlas on risk factors for type 2 diabetes: a wide-angled Mendelian randomisation study

Shuai Yuan[1], Susanna C. Larsson[1,2]

Affiliations

[1] Unit of Cardiovascular and Nutritional Epidemiology, Institute of Environmental Medicine, Karolinska Institutet, Stockholm, Sweden

[2] Department of Surgical Sciences, Uppsala University, Uppsala, Sweden

ESM Method **Pages 2-4**

ESM Table 1. Published Mendelian randomisation studies of possible risk factors for type 2 diabetes **Pages 5-6**
ESM Table 2. Information of the data sources for exposures included in this Mendelian randomisation study **Pages 7-9**
ESM Table 3. Definitions for exposures **Pages 10-15**
ESM Table 4. Power estimation **Page 16**
ESM Table 5. False discovery rate adjusted *p* values for all tested associations using DIAGRAM consortium **Pages 17-19**
ESM Table 6. Associations of 97 exposures with type 2 diabetes in Mendelian randomisation analyses **Pages 20-22**
ESM Table 7. Replication analyses using data from the FinnGen consortium for associations with *p*<0.05 in IVW analysis based on the DIAGRAM consortium **Page 23**

ESM Figure 1. Rationale and key assumptions for Mendelian randomization study **Page 24**
ESM Figure 2. Scatterplot of the alcohol-associated single nucleotide polymorphisms (SNPs) with alcohol consumption (standard deviations of log-transformed drinks/week) and type 2 diabetes (T2D) **Page 25**

ESM Method

The review of risk factors in observational study

Database: PubMed
Time: Until January 10, 2020
In total, 1360 papers
Search strategy: (((((((((Meta-Analysis[Publication Type]) OR meta-analysis[Title/Abstract]) OR meta analysis[Title/Abstract]) OR meta-analyses[Title/Abstract]) OR meta analyses[Title/Abstract]) OR Systematic Review[Publication Type]) OR systematic review[Title/Abstract])) AND ((Risk Factors[MeSH Terms]) OR risk factor[Title/Abstract])) AND (((Diabetes Mellitus, Type 2[MeSH Terms]) OR type 2 diabetes[Title/Abstract]) OR type 2 diabetes mellitus[Title/Abstract]))
We firstly deleted all replicates with the same study materials. Since we aimed to pinpoint the possible risk factors for type 2 diabetes (not a quantitative meta-analysis), we merely screened the title and abstract of the studies. The studies with the same topic were included once regardless of used materials (study base). Results from this review are presented below:

Health status	Nutrition	Lifestyle	Internal biomarker	Socioeconomic status and other factors
Hidradenitis suppurativa	Diet quality	Coffee consumption	Aldose reductase	Education
Obstructive sleep apnea	Diet pattern	Tea consumption	Fasting glucose	Income
Polycystic ovary syndrome	Dietary energy density	Alcohol consumption	Intercellular adhesion molecule-1	Occupation (long work hours, job strain, workplace bullying and violence, shift work)
Alzheimer's disease	Fatty acid consumption	Smoking	High-mobility group A1	Exposure to air pollution
Asthma	Protein consumption	Smokeless tobacco (snus)	Transforming growth factor-β1	Exposure to environmental chemicals (organochlorine pollutants, arsenic, cadmium, mercury, phthalates and bisphenol A)
Psoriasis	Isoflavones & Flavonoids consumption	Daytime napping	Thyroid-stimulating hormone & free thyroxine	Sun exposure
Psoriatic arthritis	Cholesterol consumption	Sleep (duration & short sleep)	Triglycerides	Residential proximity to major roadways
Osteoarthritis	Fibre consumption	Circadian	High-density lipoprotein cholesterol	Adverse childhood experience
Giant cell arteritis	Polyphenols consumption	Breakfast skipping	Low-density lipoprotein cholesterol	Food insecurity
Dupuytren disease	Insulinotropic amino acids consumption	Physical activity	Total cholesterol	Cardiovascular drugs (Statin therapy)
Non-alcoholic Fatty Liver Disease	Peptides consumption	Areca-nut chewing	Serum alanine aminotransferase	Depression treatment & Antipsychotics

Atopic dermatitis	Fructose consumption	Intermittent fasting	Serum aspartate aminotransferase	Spousal diabetes status
Chronic toxoplasmosis	Sucrose consumption		Serum gamma-glutamyltransferase	Hepatitis C Virus infection
Hypertriglyceridemia waist	Resveratrol consumption	**Inflammation biomarker**	Serum uric acid	Laparoscopic sleeve gastrectomy
Thyroid function	Antioxidants consumption	TNF	Serum ferritin	Bariatric surgery
Bullous skin diseases	Potato consumption	IL-1β	Fibroblast growth factor 21	
Inflammatory bowel disease	Yoghurt consumption	IL-6	Fetuin-A levels	
Rheumatoid arthritis	Egg consumption	C-reactive protein	Serum afamin	
Systemic autoimmune disorders	Vegetable consumption (citrus and cruciferous)	IL-18	Blood salicylates	
Allergic rhinitis	Soy consumption	IL-10	Lipoprotein(a)	
Food allergies	Diary consumption	Platelet mean volume, distribution width and count	Bilirubin levels	
Microalbuminuria	Pasta consumption		Haemoglobin A1c	
Microvascular dysfunction	Nut consumption	**Sex-related factor**	Metabolomics (alanine, phenylalanine, tyrosine and palmitoylcarnitine)	
Acanthosis nigricans	Fried-food consumption	Menopause	Glucokinase & glucokinase regulatory protein	
Periodontitis	White rice consumption	Age at menarche	Melatonin	
Gestational diabetes	Beverages consumption	Premature ovarian insufficiency	Apolipoprotein E	
Depression	Branched-chain amino acids	Testosterone level	Angiotensin	
Stress	Homocysteine	Parity		
Anxiety	Beta-carotene levels	Steroid sex hormones	**Obesity-related factor**	
Anorexia nervosa	Folate (vitamin B9)	Sex hormone-binding globulin	Lean body mass	
Schizophrenia	Vitamin B12	Breastfeeding	Visceral fat mass	
Bipolar disorder	Vitamin C		Birth weight	
Insomnia	25-hydroxyvitamin D		BMI	
Bulimia nervosa	Vitamin E		Serum adiponectin	

Binge eating disorder	Phylloquinone (vitamin K1)	Height
Systolic blood pressure	Vitamin K2	Hip circumference
ABO and Rh blood groups	Arsenic	Waist circumference
Preterm birth	Calcium	Waist-to-hip ratio
Cardiorespiratory fitness	Iron	Weight change
Telomere length	Magnesium	Favourable adiposity
Resting heart rate	Potassium	Childhood BMI
	Selenium	Leptin levels
	Zinc	Weight loss

Review of Mendelian randomization studies

Database: PubMed
Time: Until January 10, 2020
In total, 238 papers
Search strategy: ((((((Mendelian randomization[Title/Abstract]) OR Mendelian randomisation[Title/Abstract]) OR instrumental variable causal inference[Title/Abstract]) OR causal inference using instrumental variable[Title/Abstract]) OR causal inference using genetic variants[Title/Abstract])) AND (((Diabetes Mellitus, Type 2[MeSH Terms]) OR type 2 diabetes mellitus[Title/Abstract]) OR type 2 diabetes[Title/Abstract])
There was no replicated MR study on the same topic. We screened all titles and abstracts of included studies. We then extracted data (data in ESM Table 1) by reading the whole text (all included studies at the stage of screening were studies included finally). The detailed information on included studies is shown in ESM Table 1.

ESM Table 1. Published Mendelian randomisation studies of possible risk factors for type 2 diabetes

Exposure	Outcome GWAS	PMID	Year	SNPs	Cases	Controls	OR	95% CI	Unit	Largest T2DM GWAS
**Health status**										
Systolic blood pressure	Morris AP et al, 2012	27702834	2017	28	37293	125686	1.02	1.01-1.03	1 mmHg	NO
Telomere length	Morris AP et al, 2012	28241208	2017	11	10415	53655	1	0.84-1.20	SD	NO
Resting heart rate	Mahajan A et al, 2018	31648709	2019	Genetic score	74124	824006	1.12	1.11-1.12	10 beats/min	YES
**Nutritional factor**										
α-Linolenic acid	Mahajan A et al, 2018	31690987	2019	1	74124	824006	0.93	0.90-0.96	SD	YES
Eicosapentaenoic acid	Mahajan A et al, 2018	31690987	2019	2	74124	824006	1.08	1.03-1.12	SD	YES
Docosapentaenoic acid	Mahajan A et al, 2018	31690987	2019	2	74124	824006	1.04	1.02-1.07	SD	YES
Docosahexaenoic acid	Mahajan A et al, 2018	31690987	2019	1	74124	824006	1.04	0.94-1.15	SD	YES
Linoleic acid	Mahajan A et al, 2018	31690987	2019	3	74124	824006	0.96	0.94-0.98	SD	YES
Arachidonic acid	Mahajan A et al, 2018	31690987	2019	2	74124	824006	1.03	1.02-1.05	SD	YES
Palmitoleic acid	Mahajan A et al, 2018	31690987	2019	4	74124	824006	0.86	0.81-0.91	SD	YES
Oleic acid	Mahajan A et al, 2018	31690987	2019	1	74124	824006	0.87	0.81-0.93	SD	YES
Palmitic acid	Mahajan A et al, 2018	31690987	2019	1	74124	824006	0.98	0.87-1.09	SD	YES
Stearic acid	Mahajan A et al, 2018	31690987	2019	3	74124	824006	1.09	1.09-1.15	SD	YES
Dairy product intake	Vissers LET et al, 2019 (EPIC-InterAct)	30728219	2019	1	9686	12134	0.99	0.93-1.05	15 g/day	NO
Isoleucine	Lotta LA et al, 2016 (Morris AP et al, 2013+EPIC-InterAct+UK Biobank)	27898682	2016	4	47877	267694	1.44	1.26-1.65	SD	NO
Leucine		27898682	2016	1	47877	267694	1.85	1.41-2.42	SD	NO
Valine		27898682	2016	1	47877	267694	1.54	1.28-1.84	SD	NO
Homocysteine	Morris AP et al, 2012	26664883	2015	Genetic score	34840	114981	1.09	0.92-1.30	SD	NO
Homocysteine	Huang T et al, 2013 (17 studies)	24320691	2013	1	4011	4303	1.29	1.09-1.51	5 µmol/L	NO
25-hydroxyvitamin D	Mahajan A et al, 2018	31548248	2019	7	74124	824006	0.94	0.88-0.99	SD	YES
Beta-carotene levels	Zeggini E et al, 2008	19662379	2009	1	4549	5579	0.98	0.91-1.04	0.27 SD	NO
Vitamin B12	Morris AP et al, 2012	29982347	2018	11	34840	114981	0.96	0.71-1.30	1 SD rank-transformed	NO
Phylloquinone (vitamin K1)	Morris AP et al, 2012+UK Biobank	30352877	2019	4	69647	551336	0.93	0.89-0.97	1 ln(nmol/L)	NO
Calcium	Mahajan A et al, 2018	31548248	2019	6	74124	824006	1.05	0.85-1.28	SD	YES
Iron	Xue A et al, 2018	30759836	2019	10	62892	596424	0.89	0.81-0.98	per-unit	NO
Magnesium		30759836	2019	3	62892	596424	1.55	0.26-9.25	SD	NO
Selenium	Yarmolinsky J et al, 2018 (Morris AP et al, 2013+EPIC-InterAct+UK Biobank)	29788239	2018	4	49266	249906	1.18	0.97-1.43	114 µg/L	NO
Zinc	Xue A et al, 2018	30759836	2019	2	62892	596424	1.01	0.92-1.12	per-unit	NO
**Lifestyle factor**										
Coffee consumption [a]	Morris AP et al, 2012	27845333	2016	9	34840	114981	1.02	0.76-1.36	cups of regular-type coffee/day	NO
Smoking initiation	Mahajan A et al, 2018	31852999	2019	377	74124	824006	1.28	1.20-1.37	NA	YES
Sleep duration	Scott RA et al, 2017	30508554	2019	68	26676	132532	0.85	0.64-1.13	NA	NO

Grip strength	Mahajan A et al, 2018	30798333	2019	130	74124	824006	0.72	0.51-1.01	SD	YES
**Inflammatory factor**										
IL-1 receptor antagonist	Voight BF et al, 2010+Morris AP et al, 2012+EPIC-InterAct	25726324	2015	2	18715	61691	0.99	0.97-1.10	0.22SD	NO
IL-6 receptor	IL6 MR consortium (40 studies)	22421340	2012	1	12895	86807	0.97	0.94-1.00	15 mg/mL	NO
C-reactive protein	Morris AP et al, 2012 (stage 1)	29753585	2018	15	12171	56862	1.15	0.93-1.42	1 ln(mg/L)	NO
C-reactive protein	Morris AP et al, 2012 (stage 1)	30619477	2018	4	12171	56862	1.11	1.06-1.17	NA	NO
IL-18	Morris AP et al, 2012	31024619	2019	8 (correlated)	26488	83964	1.14	1.03-1.26	SD	NO
**Internal biomarker**										
Thyroid-stimulating hormone	Morris AP et al, 2012 (stage 1)	28323940	2017	20	12171	56862	0.91	0.78-1.07	SD	NO
Free thyroxine	Morris AP et al, 2012 (stage 1)	28323940	2017	4	12171	56862	1.10	0.94-1.30	SD	NO
HDL- cholesterol	Morris AP et al, 2012	27487401	2016	140	34840	114981	0.83	0.76-0.90	SD (16-mg/dL)	NO
LDL- cholesterol	Morris AP et al, 2012	27487401	2016	130	34840	114981	0.86	0.80-0.93	SD (38 mg/dL)	NO
Total triglyceride	Morris AP et al, 2012	27487401	2016	140	34840	114981	1.01	0.91-1.11	SD (89 mg/dL)	NO
Alanine aminotransferase	Xue A et al, 2018	31088856	2019	4	62892	596424	1.45	1.10-1.92	SD	NO
Aspartate aminotransferase	Xue A et al, 2018	31088856	2019	3	62892	596424	1.25	1.14-1.38	SD	NO
Alkaline phosphatase	Xue A et al, 2018	31088856	2019	15	62892	596424	0.91	0.86-0.97	SD	NO
γ-glutamyl transferase	Xue A et al, 2018	31088856	2019	26	62892	596424	0.92	0.80-1.06	SD	NO
Serum uric acid	Morris AP et al, 2012	26821629	2016	28	26488	83964	0.95	0.86-1.05	SD	NO
Serum ferritin	Morris AP et al, 2012 (stage 1)	26446360	2015	2	12171	56862	0.79	NA	NA	NO
Plasma fetuin-A levels	Morris AP et al, 2012 (stage 1)	29523632	2018	Genetic score	12171	56862	1.02	0.97-1.07	50 μg/mL	NO
Bilirubin levels	Abbasi A, et al, 2015 (a cohort study)	25368098	2015	1	210	3171	0.58	0.39-0.84	SD	NO
**Hormone-related factor**										
Age at menarche	Scott RA et al, 2017	31614369	2020	118	26676	132532	0.83	0.78-0.88	1 year	NO
Testosterone	UK Biobank	medRxiv	2019	81	11079	146152	1.07	0.80-1.43	0.1 nmol/L	NO
Sex hormone binding globulin	DIAGRAM Consortium, et al, 2014	26050255	2015	11	41439	103870	0.83	0.76-0.91	SD	NO
**Obesity-related factor**										
Lean body mass (men)	Mahajan A et al, 2018	30798333	2019	313	37358	428483	0.94	0.88-1.01	SD	YES
Lean body mass (women)	Mahajan A et al, 2018	30798333	2019	311	36766	395523	0.91	0.84-0.99	SD	YES
Visceral fat mass (men)	UK Biobank	31501611	2019	44	10002	150830	2.50	1.98-3.14	1 kg increase	NO
Visceral fat mass (women)	UK Biobank	31501611	2019	44	4670	154511	7.34	4.48-12.0	1 kg increase	NO
Birthweight	Morris AP et al, 2012	31539074	2019	7	34840	114918	2.79	1.90-4.20	SD	NO
BMI	UK Biobank	31821322	2019	57	13982	273412	1.31	1.11-1.53	1 kg/m^2	NO
Serum adiponectin	Voight BF et al, 2010	23835345	2013	4	15960	64731	1.06	0.84-1.33	SD	NO
Waist-to-hip ratio adj for BMI	Morris AP et al, 2012	28196256	2017	48	40530	221277	1.77	1.57-2.00	SD	NO
IGFBP-3	Morris AP et al, 2012 (stage 1)	31235487	2019	6[b]	12171	56862	1.26	1.11-1.43	SD	NO
Childhood BMI	Morris AP et al, 2012	29483184	2018	15	34840	114981	1.83	1.46-2.30	SD	NO

[a] Effect estimation was based on instrumental variables excluding body mass index-related SNPs. OR of type 2 diabetes was 1.20 (95% CI, 1.00, 1.42) based on all genome-wide significant SNPs for coffee consumption. [b] Only one SNP was associated with IGFBP-3 at the genome-wide significance level. The link of medRxiv is: https://www.medrxiv.org/content/10.1101/19005132v1.

ESM Table 2. Information of the data sources for exposures included in this Mendelian randomisation study

Risk factor	PMID	Year	Cases	Controls	Population	SNP*	SNPs excluded due to LD	Unit
**Somatic health status**								
Asthma	29273806	2018	19 954	107 715	European	16	1	Events
Atopic dermatitis	26482879	2015	18 900	84 166	European	21	0	Events
Dupuytren disease	28886342	2017	4041	8251	European	25	0	Events
Giant cell arteritis	28041642	2017	2134	9125	European	3	0	Events
Hyperthyroidism	30367059	2018	3340	49 983	European	8	0	Events
Hypothyroidism	30367059	2018	1840	49 983	European	8	0	Events
Microalbuminuria	30220432	2018	382 500	NA	European	33	0	SD
Microvascular dysfunction	26343387	2015	60 801	123 504	Mix	44	1	Events
Osteoarthritis	29559693	2018	30 727	297 191	European	8	0	Events
Periodontitis	29346566	2017	1030	9471	European	2	0	Events
	30218097	2019	5095	9908	European	2	0	Events
Polycystic ovary syndrome	30566500	2018	10 074	103 164	European	14	0	Events
Rheumatoid arthritis	24390342	2014	18 136	49 724	European	27	0	Events
Systolic blood pressure	30224653	2018	> 1 million	NA	Mix	229	11	mmHg
Telomere length	28241208	2017	9190	NA	European	16	6	SD
**Mental health status**								
Anorexia nervosa	31308545	2019	16 992	55 525	European	8	0	Events
Lifetime anxiety disorder	BioRxiv	2019	25 453	58 113	European	5	0	Events
Post-traumatic stress disorder	31594949	2019	23 212	151 447	European	2	0	Events
Schizophrenia	29483656	2018	11 260	24 542	European	143	17	Events
**Nutritional factor & Internal biomarker**								
Homocysteine	23824729	2013	44 147	NA	European	18	4	SD
Isoleucine	27898682	2016	16 596	NA	European	5	0	SD
Leucine	27898682	2016	16 597	NA	European	1	0	SD
Valine	27898682	2016	16 598	NA	European	1	0	SD
β-carotenoid (precursor to vitamin A)	19185284	2009	3941	NA	European	1	0	0.27 SD
Retinol (vitamin A)	21878437	2011	5006	NA	European	2	0	ln(ug/L)
Vitamin B6	19303062	2009	1864	NA	European	1	0	SD
Folate (vitamin B9)	23754956	2013	37 341	NA	Mix	3	1	SD
Vitamin B12	23754956	2013	45 576	NA	Mix	15	1	SD
Vitamin C	20519558	2010	15 087	NA	European	1	0	SD
Vitamin E	21729881	2011	5006	NA	European	3	0	ln(mg/L)
Copper	23720494	2013	2603	NA	European	2	0	NA
Iron	25352340	2014	48 972	NA	European	5	0	SD
Magnesium	20700443	2010	15 366	NA	European	6	0	SD
Potassium (urinary)	31409800	2019	446 238	NA	European	13	0	mmol/L
Sodium (urinary)	31409800	2019	446 237	NA	European	50	3	mmol/L

Selenium	30972295	2019	9639	NA	European	4	2	NA
Zinc	23720494	2013	2603	NA	European	3	0	NA
Thyroid-stimulating hormone	30367059	2018	72 167	NA	European	61	14	SD
Free thyroxine	30367059	2018	72 167	NA	European	31	7	SD
High-density lipoprotein cholesterol	24097068	2013	188 577	NA	Mix	71	0	SD
Low-density lipoprotein cholesterol	24097068	2013	188 577	NA	Mix	58	0	SD
Total cholesterol	24097068	2013	188 577	NA	Mix	74	0	SD
Total triglyceride	24097068	2013	188 577	NA	Mix	40	0	SD
Alanine aminotransferase	22001757	2011	61 089	NA	Mix	4	0	per 100% change
Alkaline phosphatase	22001757	2011	61 091	NA	Mix	14	0	per 100% change
γ-glutamyl transferase	22001757	2011	61 092	NA	Mix	26	0	per 100% change
Serum uric acid	31578528	2019	288 649	NA	European	123	26	1 mg/mL
Serum ferritin	25352340	2014	48 972	NA	European	8	0	SD
Fetuin-A levels	25611809	2015	2734	NA	European	1	0	SD
Lipoprotein(a)	20032323	2009	15 937	NA	European	2	0	SD
Bilirubin levels	31801373	2019	39 261	NA	European	10	0	1 mg/dL
Alanine	27005778	2016	22 569	NA	European	1	0	SD
Phenylalanine	27005778	2016	22 660	NA	European	2	0	SD
Tyrosine	27005778	2016	24 918	NA	European	1	0	SD
Haemoglobin	23222517	2012	135 367	NA	Mix	27	1	1 g/dl
**Inflammatory factor**								
TNF	NA	2016	30 912	NA	European	4	1	ln(mg/L)
C-reactive protein	30388399	2018	204 402	NA	European	58	0	ln(mg/L)
IgE	22075330	2012	6819	NA	European	3	0	ln(mg/L)
IL-1 receptor antagonist	24969107	2014	2160	NA	European	2	0	0.22 SD
IL-2 receptor subunit α	27989323	2017	8293	NA	European	1	0	SD
IL-6 receptor subunit α	29875488	2018	3301	NA	European	1	0	NA
IL-16	27989323	2017	3483	NA	European	1	0	SD
IL-17	27989323	2017	7760	NA	European	1	0	SD
IL-18	27989323	2017	3636	NA	European	3	0	SD
Mean platelet volume	27863252	2016	164 454	NA	European	294	75	SD
Platelet count	27863252	2016	166 066	NA	European	287	80	SD
Platelet distribution width	27863252	2016	164 433	NA	European	206	66	SD
Plateletcrit	27863252	2016	164 339	NA	European	272	85	SD
**Lifestyle and sleep-related factor**								
Alcohol consumption	30643251	2019	941 280	NA	European	99	16	Drinks/week
Coffee consumption	31046077	2019	375 833	NA	European	14	2	50% increase
Caffeine intake	21490707	2011	47 341	NA	European	2	0	Cubic-root transformed mg/d
Breakfast skipping	31190057	2019	193 860	NA	European	6	0	NA
Lifetime smoking	31689377	2019	462 690	NA	European	126	0	SD
Daytime napping	31409809	2019	452 071	NA	European	37	1	Daytime sleepiness

Sleep duration	30846698	2019	446 118	NA	European	78	5	Hours/day
Short sleep (<7 hours)	30846698	2019	106 192	305 742	European	27	2	Events
Long sleep (>9 hours)	30846698	2019	34 184	305 742	European	8	0	Events
Apnoea-hypopnea index	31786426	2019	1786	NA	Mix	2	0	Events/hour
Insomnia	30804565	2019	397 972	933 038	European	248	40	Events
Morningness	30696823	2019	372 765	278 530	European	351	32	Events
Restless leg syndrome	29029846	2017	15 126	95 725	European	20	0	Events
Moderate to vigorous physical activity	29899525	2018	377 234	NA	European	9	0	SD
Strenuous sports or other exercises	29899525	2018	124 842	225 650	European	6	0	≥2–3 vs. 0 days/week
Vigorous physical activity	29899525	2018	98 060	162 995	European	5	0	≥3 vs 0 days/week
Accelerometery	29899525	2018	91084	NA	European	2	0	
**Sex-hormone related factor & sex hormone**								
Age at menarche	28436984	2017	370 000	NA	Mix	389	0	1 year
Age at menopause	26414677	2015	70 000	NA	European	42	0	1 year
Testosterone levels	32042192	2020	425 097	NA	European	239	0	SD
Sex hormone binding globulin	32042192	2020	425 097	NA	European	659	0	SD
Oestradiol levels	29325096	2018	11 097	NA	European	2	0	1 pg/mL
**Obesity-related factor & education**								1 milli-gravities
Birthweight	23202124	2013	69 308	NA	European	7	0	0.1kg
Childhood BMI	26604143	2016	35 668	NA	Mix	15	0	SD
Adulthood BMI	30124842	2018	700 000	NA	European	941	280	SD
Adulthood height	30124842	2018	700 000	NA	European	3290	1574	SD
Body fat percentage	30305743	2018	500 000	NA	European	370	0	SD
Visceral fat mass	31501611	2019	400 000	NA	European	44	7	1 kilogram
Adiponectin levels	22479202	2012	45 891	NA	Mix	17	4	µg/mL
Leptin levels	26833098	2016	52 140	NA	European	5	0	ln(µg/mL)
Education	30038396	2018	1 131 881	NA	European	1271	475	SD

NA, not available; PMID, PubMed ID; SD indicates standard deviation; SNP, single-nucleotide polymorphism
All biomarkers were measured in serum or blood levels, except for sodium and potassium in urinary levels.
Source for Lifetime anxiety disorder: https://www.biorxiv.org/content/10.1101/203844v2.full
*Not all these SNPs were included in the MR analyses because some were not associated with the exposure at the genome-wide significance level ($p<5\times10^{-8}$), were in linkage disequilibrium, or not available in the type 2 diabetes dataset.

ESM Table 3. Definitions for exposures

Exposure	PubMed ID	Definition
**Somatic health status**		
Asthma	29273806	The definition of asthma was based on physicians' diagnoses and/or standardized questionnaires.
Atopic dermatitis	26482879	Clinical diagnosis
Dupuytren disease	28886342	The affected case subjects were individuals who had undergone surgical treatment for their disease.
Giant cell arteritis	28041642	The diagnosis of GCA was established according to the 1990 American College of Rheumatology classification criteria for this disease.9 In addition, the diagnosis was subsequently confirmed by either a biopsy of the temporal artery (89.83%) or arterial imaging (10.17%) consistent with GCA.
Hyperthyroidism	30367059	Hyperthyroidism cases with TSH below the reference range
Hypothyroidism	30367059	Hypothyroidism cases with TSH levels above the cohort-specific reference range
Microalbuminuria	30220432	Microalbuminuria was defined as urine ACR of 25–355 mg/g in females and 17–250 mg/g in males.
Microvascular dysfunction	26343387	Case status was defined by an inclusive CAD diagnosis (for example, myocardial infarction, acute coronary syndrome, chronic stable angina or coronary stenosis of >50%).
Osteoarthritis	29559693	We defined osteoarthritis on the basis of both self-reported status and linkage to Hospital Episode Statistics data, as well as the joint specificity of the disease (knee and/or hip).
Periodontitis	29346566	Clinical diagnosis
	30218097	Clinical diagnosis
Polycystic ovary syndrome	30566500	Cases were diagnosed with PCOS based on NIH or Rotterdam Criteria or by self-report.
Rheumatoid arthritis	24390342	All RA cases fulfilled the 1987 criteria of the American College of Rheumatology for RA diagnosis or were diagnosed with RA by a professional rheumatologist.
Systolic blood pressure	30224653	The mean SBP and DBP values from two automated or two manual blood pressure measurements
Telomere length	28241208	LTL measurement was performed by Southern blot analysis of the terminal restriction fragments, generated by the restriction enzymes *Hinf*I and *Rsa*I after verification of DNA integrity.
**Mental health status**		
Anorexia nervosa	31308545	Case definitions established a lifetime diagnosis of anorexia nervosa via hospital or register records, structured clinical interviews, or online questionnaires based on standardized criteria (Diagnostic and Statistical Manual of Mental Disorders (DSM) III-R, DSM-IV, International Classification of Diseases (ICD) 8, ICD-9 or ICD-10), whereas in the UK Biobank, cases self-reported a diagnosis of anorexia nervosa.
Lifetime anxiety disorder	BioRxiv	Cases met one of two definitions. First was self-reporting a lifetime professional diagnosis of one of the core five anxiety disorders, (generalised anxiety disorder, social phobia, panic disorder, agoraphobia or specific phobia; n=21 108). Further case was defined as meeting criteria for a likely lifetime diagnosis of DSM-IV generalised anxiety disorder based on anxiety questions from the Composite International Diagnostic Interview (CIDI) Short-form questionnaire.
Post-traumatic stress disorder	31594949	PTSD assessment was based either on lifetime (where possible) or current PTSD (i.e. including participants with a potential lifetime PTSD diagnosis as controls), and PTSD diagnosis was established using various instruments and different versions of the DSM (DSM-III-R, DSM-IV, DSM-5).
Schizophrenia	29483656	Unknown

**Nutritional factor & Internal biomarker**		
Homocysteine	23824729	Homocysteine was measured in each cohort by using one of the following methods: isotope-dilution liquid chromatography–tandem mass spectrometry, gas chromatography–coupled mass spectrometry, HPLC, or enzymatic, immune, or chemiluminescence
Isoleucine	27898682	measured using liquid chromatography coupled with tandem mass spectrometry; using gas chromatography mass spectrometry or ultra-performance liquid chromatography coupled with tandem mass spectrometry; or by nuclear magnetic resonance at 0 and 120 min during the course of an oral glucose tolerance test.
Leucine	27898682	
Valine	27898682	
β-carotenoid	19185284	Blood samples were measured via high-performance liquid chromatography.
Retinol (vitamin A)	21878437	fasting serum samples using reversed-phase liquid chromatography with diode-array UV detection
Vitamin B6	19303062	Unknown
Folate (vitamin B9)	23754956	In Inter99 serum B12 and folate were measured by a competitive chemiluminescent enzyme immunoassay as previously reported. In Health2006, serum B12 and folate were measured by chemiluminescent immunoassay.
Vitamin B12	23754956	
Vitamin C	20519558	Blood samples were taken after a minimum 6-h fast, and samples were used for assessment of circulating L-ascorbic acid.
Vitamin E	21729881	Serum alpha-tocopherol levels were measured by high-performance liquid chromatography, with a coefficient of variation (CV) of 2.2%. Gamma-tocopherol was not measured at study baseline.
Copper	23720494	Cu concentrations were measured by inductively coupled plasma mass spectrometry on a Perkin-Elmer Elan 5000 mass spectrometer or a Varian UltraMass.
Iron	25352340	Clinically and readily measurable
Magnesium	20700443	Serum magnesium levels were determined using the method described by Gindler and Heth with metallochromic dye, Calmigate [1,-[1-hydroxy-4-methyl-2-phenylazo)-2-napthol-4-sulfonic acid] in the ARIC Study, by METPATH in FHS, with a Merck Diagnostica kit (method Xylidyl blue) on an Elan Autoanalyzer (Merk) in RS, with a Xylidylblue kit on a Modular analyzer (Roche) in the KORA Study, or using a commercial colorimetric test (Roche Diagnostics, Mannheim, Germany) with a Hitachi 717 autoanalyzer in the SHIP Study.
Potassium (urinary)	31409800	Sodium and potassium concentrations in stored urine samples were measured by the ion selective electrode method (potentiometric method) using Beckman Coulter AU5400, UK Ltd.
Sodium (urinary)	31409800	
Selenium	30972295	Se and Zn concentrations were measured by inductively coupled plasma mass spectrometry on a Perkin-Elmer Elan 5000 mass spectrometer or a Varian UltraMass.
Zinc	23720494	
Thyroid-stimulating hormone	30367059	Unknown
Free thyroxine	30367059	Unknown
High-density lipoprotein cholesterol	24097068	Blood lipid levels were typically measured after >8 h of fasting. Individuals known to be on lipid-lowering medication were excluded when possible. LDL cholesterol levels were directly measured in ten studies (24% of total study individuals) and were estimated using the Friedewald formula41 in the remaining studies.
Low-density lipoprotein cholesterol	24097068	
Total cholesterol	24097068	
Total triglyceride	24097068	
Alanine aminotransferase	22001757	NMR spectroscopy on serum samples from 2,269 LOLIPOP and 4,247 NFBC1966 participants with genome-wide data to investigate the relationships of the identified loci with lipoprotein and intermediary metabolism.
Alkaline phosphatase	22001757	
γ-glutamyl transferase	22001757	
Serum uric acid	31578528	The laboratory methods were used to measure serum urate.
Serum ferritin	25352340	Clinically and readily measurable

Fetuin-A levels	25611809	Plasma concentrations of fetuin- A were measured by enzyme- linked immunosorbent assays (Human Fetuin-A ELISA; BioVendor) in the laboratory of Professor Pischon, Molecular Epidemiology Group, Max Delbrück Center for Molecular Medicine (MDC), Berlin- Buch, Germany.
Lipoprotein(a)	20032323	Lp(a) lipoprotein was measured by means of one latex-enhanced immunoturbidimetric assay (Immuno)16 in samples from case subjects that had been obtained in study clinics. In addition, Lp(a) lipoprotein was measured in a random subgroup of case subjects and control subjects with the use of a second latex-enhanced immunoturbidimetric assay (Randox Laboratories) on an ADVIA 1800 autoanalyzer (Siemens).
Bilirubin levels	31801373	Serum total bilirubin levels (both direct and indirect bilirubin) were measured through automated biochemical profiling, and the unit of bilirubin concentration was milligram per deciliter.
Alanine	27005778	The quantitative high-throughput NMR metabolomics platform, used to quantify human blood metabolites, was applied
Phenylalanine	27005778	
Tyrosine	27005778	
Hemoglobin	23222517	Unknown
**Inflammatory factor**		
TNF	NA	Each study typically collected venous blood samples from their participants frozen as either serum or plasma and stored below -80°C until the time of measurement. Serum or plasma levels of TNF-a were measured using various types of immunoassays and expressed as pg/ml.
C-reactive protein	30388399	serum CRP in mg/L by was measured using standard laboratory techniques and transformed the values by natural log.
IgE	22075330	Total IgE concentrations were measured with the FEIA CAP system (Pharmacia, Freiburg, Germany).
IL-1 receptor antagonist	24969107	IL-1RA serum concentrations were measured using the Quantikine ELISA Kit (R&D Systems, Wiesbaden, Germany).
IL-2 receptor subunit α	27989323	A total of 48 cytokines were measured by using Bio-Rad's premixed Bio-Plex Pro Human Cytokine 27-plex Assay and 21-plex Assay, and Bio-Plex 200 reader with Bio-Plex 6.0 software.
IL-6 receptor subunit α	29875488	A multiplexed, aptamer-based approach (SOMAscan assay) was used to measure the relative concentrations of 3,622 plasma proteins or protein complexes assayed using 4,034 modified aptamers.
IL-16	27989323	A total of 48 cytokines were measured by using Bio-Rad's premixed Bio-Plex Pro Human Cytokine 27-plex Assay and 21-plex Assay, and Bio-Plex 200 reader with Bio-Plex 6.0 software.
IL-17	27989323	
IL-18	27989323	
Mean platelet volume	27863252	Full blood counts were measured in UK Biobank and INTERVAL study participants using clinical hematology analyzers at the centralized processing laboratory of UK Biocenter (Stockport, UK).
Platelet count	27863252	
Platelet distribution width	27863252	
Plateletcrit	27863252	
**Lifestyle and sleep-related factor**		
Alcohol consumption	30643251	Questionnaire survey, drinks per week
Coffee consumption	31046077	Coffee intake was collected using a 24-hour recall questionnaire (Oxford WebQ) in a subset of UK Biobank participants. Researchers used the mean intake from participants who completed at least two dietary recalls.
Caffeine intake	21490707	Intake was assessed using a validated semi-quantitative food frequency questionnaire (FFQ). For each item, participants were asked how often, on average, they had consumed a specified amount of each beverage or food over the past year. The participants could choose from nine frequency categories (never, 1–3 per month, 1 per week, 2–4 per week, 5–6 per week, 1 per day, 2–3 per day, 4–5 per day and 6 or more per day). Intakes of nutrients

		and caffeine were calculated using US Department of Agriculture food composition sources. In these calculations, researchers assumed that the content of caffeine was 137 mg per cup of coffee, 47 mg per cup of tea, 46 mg per can or bottle of cola or other caffeinated carbonated beverage, and 7 mg per 1 oz serving of chocolate candy. Researchers assessed the total intake of caffeine by summing the caffeine content for the specified amount of each food multiplied by a weight proportional to the frequency of its use.
Breakfast skipping	31190057	The breakfast cereal–skipping data from 24-h recalls from the UK Biobank
Lifetime smoking	31689377	Smoking measures available in the UK Biobank were self-reported and collected at initial assessment. They included: smoking status, age at initiation in years, age at cessation in years and number of cigarettes smoked per day. The lifetime smoking index was constructed based on the method outlined by Leffondré, Abrahamowicz, Xiao, and Siemiatyck (2006).
Daytime napping	31409809	Self-reported daytime sleepiness was ascertained in the UK Biobank using the question “How likely are you to dose off or fall asleep during the daytime when you don’t mean to? (e.g. when working, reading or driving)” with the response options of “Never/rarely”, “sometimes”, “often”, “all of the time”, “do not know”, and “prefer not to answer”. Participants reporting “do not know” and “prefer not to answer” were set to missing. Other responses were coded continuously as 1 to 4 corresponding to the severity of daytime sleepiness.
Sleep duration<br>Short sleep (<7 hours)<br>Long sleep (>9 hours)	30846698<br>30846698<br>30846698	Participants were asked: About how many hours sleep do you get in every 24 h? (please include naps), with responses in hour increments. Sleep duration was treated as a continuous variable and also categorized as either short (6 h or less), normal (7 or 8 h), or long (9 h or more) sleep duration. Extreme responses of less than 3 h or more than 18 h were excluded17 and Do not know or Prefer not to answer responses were set to missing. Participants who self-reported any sleep medication (see Supplementary Method 1) were excluded.
Apnea-hypopnea index	31786426	The AHI was defined as the total number of apneas and hypopneas per hour of sleep. The delta-AHI is the increase/decrease of AHI across eight years as shown in the formula below: delta-AHI = AHI follow-up – AHI baseline OSA was defined according to AHI. Researchers elected to define OSA as AHI≥ 15 events/hour, a cutoff point that presents more solid evidence of associated outcomes. Individuals with incident OSA were those with an AHI < 15/h at baseline, which presented an AH≥15h at follow-up. Individuals that did not develop OSA (controls) were those with an AHI ≤ 15h at both baseline and follow-up studies.
Insomnia	30804565	Insomnia complaints were measured using questionnaire data; an independent sample (the Netherlands Sleep Register)12, which gives access to similar question data, as well as clinical interviews assessing insomnia disorder, was used to validate the specific questions so that they were good proxies of insomnia disorder.
Morningness	30696823	Responses to two identical questions (“Are you naturally a night person or a morning person?”) were used to define the dichotomous morning person phenotype in the 23andMe cohort, with one question having a wider selection of neutral options. For the first instance, the possible answers were “Night owl”, “Early bird” and “Neither”, and for the second “Night person”, “Morning person”, “Neither”, “It depends” and “I’m not sure”. Individuals with discordant or neutral responses to both were excluded. For those with one neutral and one non-neutral response, their non-neutral response was used to define their phenotype. Morning people were coded as 1 (cases; N = 120,478) and evening people were coded as 0 (controls; N = 127,622). The UK Biobank collected a single self-reported measure of Chronotype (“Morning/evening person (chronotype)”; data-field 1180). Participants were prompted to answer the question “Do you consider yourself to be?” with one of six possible answers: “Definitely a ‘morning’ person”, “More a ‘morning’ than ‘evening’ person”, “More an ‘evening’ than a ‘morning’ person”, “Definitely an ‘evening’ person”, “Do not know” or “Prefer not to answer”, which we coded as 2, 1, −1, −2, 0 and missing, respectively.

Restless leg syndrome	29029846	People with restless legs syndrome were recruited in specialist outpatient clinics for movement disorders and in sleep units. Restless legs syndrome was diagnosed in face-to-face interviews by an expert neurologist, based on the International Restless Legs Syndrome Study Group diagnostic criteria
Moderate to vigorous physical activity	29899525	Moderate-to-vigorous PA (MVPA) was calculated by taking the sum of total minutes/week of MPA multiplied by four and the total number of VPA minutes/week multiplied by eight, corresponding to their metabolic equivalents.
Strenuous sports or other exercises	29899525	In the last 4 weeks did you spend any time doing the following?" and follow-up questions assessing the frequency and typical duration of "strenuous sports" and of "other exercises". The possible responses to the initial question were: 'walking for pleasure', 'other exercises', 'strenuous sports', 'light DIY', 'heavy DIY', 'none of the above', and 'prefer not to answer'. Researchers identified individuals spending 2–3 days/week or more doing strenuous sports or other exercises (SSOE), for a duration of 15–30 min or greater.
Vigorous physical activity	29899525	For vigorous PA (VPA), participants were asked: "In a typical WEEK, how many days did you do 10 min or more of vigorous physical activity? (These are activities that make you sweat or breathe hard such as fast cycling, aerobics, heavy lifting)". For each of these questions, those who indicated 1 or more such days were then asked "How many minutes did you usually spend doing moderate/vigorous activities on a typical DAY". Participants were asked to include activities performed for work, leisure, travel and around the house. We excluded individuals who selected "prefer not to answer" or "do not know" on the above questions, those reporting not being able to walk, and individuals reporting more than 16 h of either MPA or VPA per day. Those reporting >3 h/day of VPA or MPA were recoded to 3 h, as recommended.
**Sex-related factor**		
Age at menarche	28436984	Age at menarche in women. Age at voice breaking in men (23andme: How old were you when your voice began to crack/deepen? UKBB: When did your voice break?)
Age at menopause	26414677	ANM was self-reported and defined as the age at last naturally occurring menstrual period followed by at least 12 consecutive months of amenorrhea.
Testosterone levels	32042192	Unknown
Sex hormone binding globulin	32042192	Unknown
Estradiol levels	29325096	In six discovery cohorts (FHS, GOOD, MrOS Sweden Gothenburg, MrOS Sweden Malmo¨ , andMrOs United States), measurements were performed using either GC-MS or liquid chromatography tandem mass spectrometry. In the remaining discovery cohorts (LURIC, Invecchiare in Chianti, Multi-Ethnic Study of Atherosclerosis, and RS1) measurements were performed using immunoassays. In the replication cohort (EMAS), E2 was measured using the GC-MS technique.
**Obesity-related factor & education**		
Birthweight	23202124	Birth weight was measured by trained personnel in some studies but in others was self-reported in adulthood.
Childhood BMI	26604143	Childhood obesity cases were defined as having a BMI ≥ 95th percentile, whereas childhood normal weight controls were defined as having a BMI < 50th percentile.
Adulthood BMI	30124842	Self-reported and measured values
Adulthood height	30124842	
Body fat percentage	30305743	Body composition estimation by impedance measurement.

Visceral fat mass	31501611	Two UKBB subcohorts were constructed: one training dataset with VAT mass measured by DXA (instance 2; n = 5,109), to which the prediction models were calibrated; and one application dataset (instances 0 and 1; n = 502,638), in which VAT mass was predicted using the calibrated prediction models.
Adiponectin levels	22479202	Adiponectin levels were measured using ELISA or RIA methods.
Leptin levels	26833098	Circulating levels of leptin

ESM Table 4. Power estimation

Effect size (odds ratio)	Variance explained by used SNPs							
	**0.001**	**0.005**	**0.01**	**0.02**	**0.05**	**0.10**	**0.15**	**0.2**
0.1	1.00	1.00	1.00	1.00	1.00	1.00	1.00	1.00
0.3	1.00	1.00	1.00	1.00	1.00	1.00	1.00	1.00
0.5	0.99	1.00	1.00	1.00	1.00	1.00	1.00	1.00
0.7	0.72	1.00	1.00	1.00	1.00	1.00	1.00	1.00
0.8	0.39	0.96	1.00	1.00	1.00	1.00	1.00	1.00
0.9	0.13	0.46	0.75	0.96	1.00	1.00	1.00	1.00
1.1	0.13	0.45	0.73	0.96	1.00	1.00	1.00	1.00
1.2	0.39	0.95	1.00	1.00	1.00	1.00	1.00	1.00
1.3	0.68	1.00	1.00	1.00	1.00	1.00	1.00	1.00
1.5	0.98	1.00	1.00	1.00	1.00	1.00	1.00	1.00
1.7	1.00	1.00	1.00	1.00	1.00	1.00	1.00	1.00
1.9	1.00	1.00	1.00	1.00	1.00	1.00	1.00	1.00
2.0	1.00	1.00	1.00	1.00	1.00	1.00	1.00	1.00
3.0	1.00	1.00	1.00	1.00	1.00	1.00	1.00	1.00

The power estimation was based on 74124 type 2 diabetes cases and 824006 controls and we assumed type 1 error rate as 0.05. The webtool power calculator: https://shiny.cnsgenomics.com/mRnd/.

ESM Table 5. False discovery rate adjusted *p* values for all tested associations using DIAGRAM consortium

Risk factor	Original *p* value	Critical Value	Benjamini-Hochberg adjusted *p* value	Significant using an FDR of 0.05?
Adulthood BMI	1.40E-44	5.21E-04	1.34E-42	Yes
Alanine	6.50E-25	0.001	3.12E-23	Yes
Body fat percentage	3.40E-23	0.002	1.09E-21	Yes
Visceral fat mass	2.20E-20	0.002	5.28E-19	Yes
Sex hormone binding globulin	9.39E-14	0.003	1.80E-12	Yes
Age at menarche	1.30E-12	0.003	2.08E-11	Yes
Systolic blood pressure	2.10E-10	0.004	2.88E-09	Yes
Insomnia	1.00E-09	0.004	1.20E-08	Yes
Lifetime smoking	4.00E-08	0.005	4.27E-07	Yes
Isoleucine	8.73E-08	0.005	8.38E-07	Yes
Childhood body mass index	3.40E-06	0.006	2.97E-05	Yes
Caffeine intake	1.00E-05	0.006	8.00E-05	Yes
Adulthood height	4.00E-04	0.007	0.003	Yes
Leucine	0.001	0.007	0.007	Yes
Valine	0.001	0.008	0.006	Yes
Testosterone levels	0.002	0.008	0.012	Yes
High-density lipoprotein cholesterol	0.002	0.009	0.011	Yes
Birthweight	0.004	0.009	0.021	Yes
Total cholesterol	0.005	0.010	0.025	Yes
Alanine aminotransferase	0.009	0.010	0.043	Yes
Phenylalanine	0.015	0.011	0.069	No
Coffee consumption	0.017	0.011	0.074	No
Tyrosine	0.019	0.012	0.079	No
IL-6 receptor subunit α	0.021	0.013	0.084	No
Sodium (urinary)	0.024	0.013	0.092	No
Selenium	0.025	0.014	0.092	No
IL-1 receptor antagonist	0.034	0.014	0.121	No
Folate (vitamin B9)	0.038	0.015	0.130	No
Low-density lipoprotein cholesterol	0.044	0.015	0.146	No
Giant cell arteritis	0.048	0.016	0.154	No
Fetuin-A levels	0.049	0.016	0.152	No
Periodontitis	0.054	0.017	0.162	No
IL-16	0.056	0.017	0.163	No
Iron	0.074	0.018	0.209	No
Apnoea-hypopnea index	0.081	0.018	0.222	No
Free thyroxine	0.099	0.019	0.264	No
Anorexia nervosa	0.100	0.019	0.259	No

Lipoprotein(a)	0.110	0.020	0.278	No
Breakfast skipping	0.129	0.020	0.318	No
Circulating adiponectin	0.144	0.021	0.346	No
Polycystic ovary syndrome	0.145	0.021	0.340	No
Atopic dermatitis	0.145	0.022	0.331	No
Vigorous physical activity	0.156	0.022	0.348	No
Tumour necrosis factor	0.164	0.023	0.358	No
IgE	0.198	0.023	0.422	No
IL-2 receptor subunit α	0.203	0.024	0.424	No
Daytime napping	0.205	0.024	0.419	No
Oestradiol levels	0.210	0.025	0.420	No
Homocysteine	0.225	0.026	0.441	No
Sleep duration	0.234	0.026	0.449	No
Copper	0.238	0.027	0.448	No
Microalbuminuria	0.241	0.027	0.445	No
Short sleep (<7 h)	0.247	0.028	0.447	No
Plateletcrit	0.273	0.028	0.485	No
Vitamin B6	0.295	0.029	0.515	No
Strenuous sports or other exercises	0.306	0.029	0.525	No
Age at menopause	0.312	0.030	0.525	No
Mean platelet volume	0.317	0.030	0.525	No
Magnesium	0.347	0.031	0.565	No
Dupuytren disease	0.352	0.031	0.563	No
Osteoarthritis	0.357	0.032	0.562	No
Post-traumatic stress disorder	0.360	0.032	0.557	No
Retinol (vitamin A)	0.364	0.033	0.555	No
β-carotenoid (precursor to vitamin A)	0.382	0.033	0.573	No
Lifetime anxiety disorder	0.388	0.034	0.573	No
Long sleep (>9 h)	0.389	0.034	0.566	No
Thyroid-stimulating hormone	0.396	0.035	0.567	No
Accelerometery	0.408	0.035	5.79E-01	No
Microvascular dysfunction	0.414	0.035	0.584	No
IL-17	0.415	0.036	0.577	No
Potassium (urinary)	0.415	0.036	0.569	No
Vitamin E	0.419	0.037	0.567	No
Bilirubin levels	0.423	0.038	0.564	No
Restless leg syndrome	0.430	0.038	0.565	No
Alkaline phosphatase	0.469	0.039	0.608	No
Platelet count	0.470	0.039	0.602	No
Rheumatoid arthritis	0.481	0.040	0.608	No
Telomere length	0.495	0.040	0.617	No

Moderate to vigorous physical activity	0.525	0.041	0.646	No
Platelet distribution width	0.613	0.041	0.745	No
Alcohol consumption	0.631	0.042	0.757	No
Serum ferritin	0.634	0.042	0.751	No
Asthma	0.647	0.043	0.757	No
Total triglyceride	0.756	0.043	0.874	No
Vitamin B12	0.762	0.044	0.871	No
Haemoglobin	0.801	0.044	0.905	No
Hyperthyroidism	0.820	0.045	0.915	No
Hypothyroidism	0.821	0.045	0.906	No
C-reactive protein	0.833	0.046	0.909	No
Serum uric acid	0.876	0.046	0.945	No
Leptin levels	0.877	0.047	0.935	No
γ-glutamyl transferase	0.897	0.047	0.946	No
Morningness	0.910	0.048	0.950	No
Zinc	0.923	0.048	0.953	No
Schizophrenia	0.944	0.049	0.964	No
Vitamin C	0.973	0.049	0.983	No
IL-18	0.974	0.050	0.974	No

The False discovery rate estimation is based on the Benjamini-Hochberg method to identify which values remain significant at an FDR of choosing, when adjusting for multiple testing (https://tools.carbocation.com/FDR).

ESM Table 6. Associations of 97 exposures with type 2 diabetes in Mendelian randomisation analyses

Risk factor	Used SNPs	F	I^2 [a]	IVW model [b] OR	IVW model 95% CI	IVW model *p* value	Weighted median model [c] OR	Weighted median model 95% CI	Weighted median model *p* value	MR-Egger OR	MR-Egger 95% CI	MR-Egger *p* value	*p* value for pleiotropy [d]
**Somatic health status**													
Asthma	15	69.8	83	1.01	0.94, 1.10	0.647	0.97	0.92, 1.02	0.217	1.35	0.99, 1.84	0.055	0.063
Atopic dermatitis	20	45.7	69	1.05	0.98, 1.11	0.145	1.06	1.00, 1.13	0.044	1.03	0.87, 1.23	0.7	0.903
Dupuytren disease	25	113.6	74	1.01	0.99, 1.03	0.352	1.00	0.99, 1.02	0.719	0.99	0.92, 1.06	0.714	0.477
Giant cell arteritis	3	117.0	91	1.07	1.00, 1.14	0.048	1.07	1.05, 1.10	2.8E-8	1.19	1.11, 1.29	5.7E-6	0.002
Hyperthyroidism	8	46.4	77	1.00	0.95, 1.04	0.820	1.01	0.98, 1.04	0.488	1.19	0.99, 1.43	0.069	0.056
Hypothyroidism	8	46.9	67	0.99	0.95, 1.04	0.821	0.99	0.95, 1.03	0.698	0.93	0.76, 1.15	0.501	0.522
Microalbuminuria	33	77.8	85	1.26	0.86, 1.85	0.241	1.21	0.96, 1.52	0.111	1.16	0.65, 2.05	0.624	0.691
Microvascular dysfunction	41	67.1	86	1.03	0.96, 1.10	0.414	1.06	1.00, 1.12	0.030	1.01	0.85, 1.21	0.872	0.852
Osteoarthritis	8	34.7	82	0.94	0.83, 1.07	0.357	0.99	0.91, 1.07	0.781	1.13	0.78, 1.63	0.526	0.309
Periodontitis	4	31.7	0	1.03	1.00, 1.06	0.054	1.03	1.00, 1.07	0.063	1.19	0.93, 1.53	0.172	0.253
Polycystic ovary syndrome	14	40.8	56	0.96	0.92, 1.01	0.145	0.95	0.91, 1.00	0.065	1.02	0.81, 1.29	0.858	0.621
Rheumatoid arthritis	27	34.4	78	0.98	0.92, 1.04	0.481	0.99	0.95, 1.04	0.734	0.92	0.76, 1.12	0.413	0.524
Systolic blood pressure	219	46.1	83	1.39	1.26, 1.54	2.1E-10	1.28	1.19, 1.38	3.5E-11	1.72	1.10, 2.68	0.017	0.346
Telomere length	10	70.8	0	1.02	0.96, 1.08	0.495	1.02	0.95, 1.10	0.547	1.19	0.91, 1.55	0.200	0.247
**Mental health status**													
Anorexia nervosa	8	53.8	66	0.92	0.84, 1.02	0.100	0.97	0.90, 1.05	0.493	1.08	0.75, 1.56	0.689	0.396
Lifetime anxiety disorder	5	42.6	83	1.05	0.94, 1.18	0.388	1.12	1.03, 1.21	0.002	1.12	0.21, 6.12	0.893	0.941
Post-traumatic stress disorder	2	34.4	-	0.96	0.87, 1.05	0.360	-	-	-	-	-	-	-
Schizophrenia	111	51.3	71	1.00	0.97, 1.04	0.944	0.98	0.95, 1.01	0.241	0.97	0.82, 1.14	0.694	0.677
**Nutritional factor & internal biomarker**													
β-carotenoid (precursor to vitamin A)	1	98.6	-	0.96	0.89, 1.05	0.382	-	-	-	-	-	-	-
Retinol (vitamin A)	2	56.9	-	1.15	0.85, 1.56	0.364	-	-	-	-	-	-	-
Vitamin B6	1	26.8	-	1.00	0.99, 1.00	0.295	-	-	-	-	-	-	-
Folate (vitamin B9)	2	83.7	-	0.88	0.78, 0.99	0.038	-	-	-	-	-	-	-
Vitamin B12	12	746.2	1	0.99	0.95, 1.04	0.762	1.00	0.95, 1.06	0.918	0.98	0.89, 1.07	0.595	0.654
Vitamin C	1	27.1	-	1.00	0.99, 1.01	0.973	-	-	-	-	-	-	-
Vitamin E	3	11.4	63	1.21	0.76, 1.93	0.419	1.30	0.80, 2.12	0.294	10.1	0.78, 130	0.076	0.101
Copper	2	62.7	-	1.03	0.98, 1.07	0.238	-	-	-	-	-	-	-
Iron	5	331.4	51	1.06	0.99, 1.13	0.074	1.06	1.00, 1.13	0.040	1.14	1.02, 1.26	0.015	0.116
Magnesium	6	58.8	75	1.08	0.92, 1.26	0.347	1.09	0.96, 1.24	0.165	1.51	1.00, 2.27	0.048	0.088
Potassium (urinary)	13	44.1	71	0.71	0.32, 1.61	0.415	0.75	0.36, 1.54	0.429	0.10	0.00, 16.3	0.374	0.442
Sodium (urinary)	47	48.1	94	2.69	1.14, 6.34	0.024	3.33	2.11, 5.27	1.5E-7	0.26	0.01, 8.07	0.446	0.170
Selenium	2	81.1	-	1.05	1.01, 1.10	0.025	-	-	-	-	-	-	0.061
Zinc	2	59.7	-	1.00	0.96, 1.04	0.923	-	-	-	-	-	-	-

Thyroid-stimulating hormone	46	82.9	90	0.96	0.87, 1.06	0.396	0.95	0.91, 1.00	0.050	0.90	0.70, 1.15	0.384	0.562
Free thyroxine	23	65.4	57	0.94	0.88, 1.01	0.099	1.01	0.94, 1.08	0.849	1.14	1.00, 1.31	0.053	0.002
High-density lipoprotein cholesterol	69	157.2	95	0.78	0.67, 0.91	0.002	1.00	0.94, 1.06	0.883	1.07	0.86, 1.32	0.54	<0.001
Low-density lipoprotein cholesterol	58	162.5	88	0.91	0.83, 1.00	0.044	0.89	0.84, 0.94	9.57E-05	0.82	0.7, 0.96	0.012	0.107
Total cholesterol	74	139.1	89	0.87	0.79, 0.96	0.005	0.87	0.81, 0.94	1.18E-04	0.78	0.65, 0.93	0.006	0.156
Total triglyceride	40	159.5	96	1.04	0.83, 1.29	0.756	1.08	1.00, 1.18	0.048	0.77	0.57, 1.05	0.094	0.011
Lipoprotein(a)	2	575.3	-	1.02	0.99, 1.03	0.110	-	-	-	-	-	-	-
Alanine aminotransferase	4	104.7	78	1.02	1.00, 1.03	0.009	1.02	1.01, 1.03	1.74E-05	1.04	1.02, 1.06	6.7E-6	0.006
Alkaline phosphatase	14	71.2	88	1.00	0.98, 1.01	0.469	1.00	0.99, 1.00	0.348	0.99	0.96, 1.01	0.231	0.333
γ-glutamyl transferase	26	188.3	91	1.00	0.99, 1.01	0.897	1.00	1.00, 1.01	0.295	1.01	0.99, 1.04	0.307	0.270
Serum uric acid	97	145.4	97	1.01	0.87, 1.18	0.876	1.02	0.98, 1.06	0.433	0.97	0.78, 1.22	0.817	0.639
Serum ferritin	8	69.8	89	1.06	0.83, 1.36	0.634	1.17	1.04, 1.31	0.009	1.45	0.96, 2.21	0.079	0.088
Fetuin-A levels	1	1331.8	-	1.02	1.00, 1.05	0.049	-	-	-	-	-	-	-
Bilirubin levels	10	190.0	67	1.10	0.87, 1.38	0.423	0.96	0.88, 1.04	0.302	0.82	0.46, 1.46	0.503	0.607
Homocysteine	13	89.5	80	1.09	0.95, 1.25	0.225	0.96	0.87, 1.05	0.374	0.96	0.63, 1.46	0.836	0.521
Isoleucine	4	36.4	0	1.26	1.16, 1.37	8.73E-08	0.98	0.76, 1.26	0.887	1.48	0.83, 2.64	0.179	0.283
Leucine	1	37.7	-	1.28	1.10, 1.49	0.001	-	-	-	-	-	-	-
Valine	1	54.3	-	1.23	1.08, 1.39	0.001	-	-	-	-	-	-	-
Alanine	1	100.0	-	0.51	0.45, 0.58	6.50E-25	-	-	-	-	-	-	-
Phenylalanine	2	72.7	-	1.15	1.03, 1.29	0.015	-	-	-	-	-	-	-
Tyrosine	1	100.0	-	0.86	0.76, 0.98	0.019	-	-	-	-	-	-	-
Haemoglobin	19	50.8	87	0.98	0.81, 1.18	0.801	1.02	0.90, 1.16	0.721	1.22	0.72, 2.07	0.452	0.367
**Inflammatory factor**													
Tumour necrosis factor	3	33.8	61	1.31	0.90, 1.91	0.164	1.31	0.93, 1.84	0.117	0.41	0.14, 1.21	0.107	0.031
C-reactive protein	57	165.8	96	1.02	0.86, 1.20	0.833	0.98	0.94, 1.02	0.358	0.96	0.75, 1.23	0.751	0.774
IgE	3	80.6	0	1.04	0.98, 1.11	0.198	0.97	0.90, 1.04	0.382	1.16	0.81, 1.65	0.420	0.532
IL-1 receptor antagonist	2	78.9	-	1.13	1.01, 1.27	0.034	-	-	-	-	-	-	-
IL-2 receptor subunit α	1	164.2	-	0.98	0.94, 1.01	0.203	-	-	-	-	-	-	-
IL-6 receptor subunit α	1	5040.4	-	0.99	0.98, 1.00	0.021	-	-	-	-	-	-	-
IL-16	1	133.1	-	0.97	0.94, 1.00	0.056	-	-	-	-	-	-	-
IL-17	1	40.4	-	1.05	0.93, 1.18	0.415	-	-	-	-	-	-	-
IL-18	3	80.3	0	1.00	0.96, 1.04	0.974	1.04	0.97, 1.11	0.262	1.09	0.6, 1.96	0.776	0.553
Mean platelet volume	284	234.7	60	0.99	0.96, 1.01	0.317	0.98	0.95, 1.02	0.394	0.99	0.95, 1.04	0.716	0.748
Platelet count	193	133.4	82	0.98	0.92, 1.04	0.470	1.00	0.95, 1.04	0.891	1.02	0.91, 1.15	0.718	0.415
Platelet distribution width	134	150.5	64	1.01	0.97, 1.06	0.613	1.02	0.97, 1.07	0.420	1.04	0.95, 1.13	0.370	0.458
Plateletcrit	173	120.6	84	0.96	0.90,1.03	0.273	0.98	0.93, 1.03	0.456	1.06	0.93, 1.22	0.371	0.098
**Lifestyle and sleep-related factor**													
Alcohol consumption	83	78.7	84	1.08	0.80, 1.45	0.631	1.46	1.16, 1.83	0.001	2.27	1.30, 3.93	0.004	0.002

Coffee consumption	12	159.5	94	1.59	1.09, 2.32	0.017	1.29	1.15, 1.46	1.8E-5	0.96	0.49, 1.90	0.916	0.091
Caffeine intake	2	69.4	-	1.17	1.09, 1.25	1.0E-5	-	-	-	-	-	-	-
Breakfast skipping	6	39.0	81	1.72	0.85, 3.46	0.129	2.34	1.41, 3.89	0.001	12.9	1.10, 150	0.042	0.099
Lifetime smoking	126	48.8	76	1.61	1.36, 1.91	4.0E-8	1.52	1.30, 1.78	6.0E-8	0.62	0.32, 1.20	0.156	0.004
Daytime napping	35	42.6	87	1.77	0.73, 4.24	0.205	2.23	1.22, 4.09	0.009	26.1	0.63, 1074	0.086	0.144
Sleep duration	73	39.9	88	0.83	0.62, 1.12	0.234	0.97	0.80, 1.17	0.741	0.93	0.30, 2.88	0.899	0.847
Short sleep (<7 h)	25	25.8	85	1.14	0.92, 1.41	0.247	1.10	0.95, 1.28	0.216	1.03	0.43, 2.45	0.945	0.821
Long sleep (>9 h)	8	28.6	97	0.79	0.47, 1.34	0.389	0.98	0.86, 1.11	0.711	2.11	0.71, 6.31	0.179	0.054
Apnoea-hypopnea index	2	32.1	-	1.05	0.99, 1.12	0.081	-	-	-	-	-	-	-
Insomnia	207	42.5	79	1.17	1.11, 1.23	1.0E-9	1.15	1.11, 1.20	1.1E-11	1.29	1.05, 1.57	0.013	0.315
Morningness	317	54.3	77	1.00	0.94, 1.05	0.910	0.97	0.92, 1.02	0.188	1.09	0.93, 1.27	0.294	0.245
Restless leg syndrome	20	697.6	62	1.01	0.98, 1.05	0.430	1.03	1.00, 1.07	0.075	1.02	0.95, 1.09	0.572	0.854
Moderate to vigorous physical activity	8	48.0	92	0.69	0.22, 2.14	0.525	1.08	0.67, 1.75	0.753	0.00	0.00, 0.01	1.2E-5	<0.001
Strenuous sports or other exercises	6	18.6	90	0.77	0.47, 1.27	0.306	0.87	0.69, 1.11	0.272	0.92	0.13, 6.71	0.937	0.854
Vigorous physical activity	5	18.0	0	0.90	0.78, 1.04	0.156	0.90	0.75, 1.06	0.193	0.82	0.28, 2.41	0.718	0.862
Accelerometery	2	18.1	16.9	0.98	0.95, 1.02	0.408	-	-	-	-	-	-	-
**Sex-related factor**													
Age at menarche	329	67.1	80	0.84	0.80, 0.88	1.3E-12	0.90	0.86, 0.93	3.7E-07	0.93	0.82, 1.06	0.291	0.071
Age at menopause	42	79.0	79	0.99	0.96, 1.01	0.312	0.99	0.97, 1.01	0.171	0.99	0.93, 1.04	0.648	0.982
Testosterone levels	209	92.3	87	0.72	0.59, 0.89	0.002	0.88	0.75, 1.03	0.125	1.22	0.87, 1.72	0.257	<0.001
Sex hormone binding globulin	477	197.6	89	0.55	0.47, 0.64	9.39E-14	0.75	0.67, 0.84	1.42E-06	0.9	0.73, 1.11	0.317	<0.001
Oestradiol levels	1	83.0	-	0.99	0.87, 1.13	0.210	-	-	-	-	-	-	-
**Obesity-related factor& education**													
Birthweight	7	73.9	98	0.79	0.67, 0.92	0.004	0.84	0.79, 0.90	9.7E-07	17.3	0.00, 2.3E6	0.636	0.608
Childhood BMI	15	57.9	95	1.87	1.44, 2.44	3.4E-06	1.79	1.59, 2.01	1.9E-22	2.24	0.78, 6.46	0.133	0.726
Adulthood BMI	661	88.4	90	1.89	1.73, 2.07	1.4E-44	1.68	1.55, 1.82	1.2E-35	1.29	1.10, 1.52	0.002	<0.001
Adulthood height	1716	235.8	72	0.95	0.92, 0.98	4.0E-4	0.95	0.92, 0.98	0.008	0.96	0.91, 1.00	0.046	0.767
Body fat percentage	370	49.4	93	2.07	1.79, 2.39	3.4E-23	2.58	2.39, 2.78	1.0E-131	2.76	1.63, 4.66	1.5E-4	0.265
Visceral fat mass	36	44.2	88	2.63	2.14, 3.23	2.2E-20	2.70	2.32, 3.15	1.8E-36	5.35	2.74, 10.4	8.8E-7	0.030
Circulating adiponectin	13	107.1	93	0.82	0.63, 1.07	0.144	0.99	0.90, 1.09	0.831	1.16	0.87, 1.55	0.300	0.001
Leptin levels	5	35.5	98	1.13	0.24, 5.37	0.877	1.06	0.75, 1.51	0.724	160	0.00, 3440	0.418	0.425

CI indicates confidence interval; IVW, inverse-variance weighted; OR, odds ratio;

[a] I^2 indicates the heterogeneity among used SNPs for each exposure.

[b] For traits with ≥3 SNPs, the results were based on IVW model with random effects, and for traits with <3 SNPs, the results were based on IVW model with fixed effects.

[c] Estimates were not available for traits with less than 3 SNPs.

[d] *P* for the intercept in the MR-Egger regression.

ESM Table 7. Replication analyses using data from the FinnGen consortium for associations with *p*<0.05 in IVW analysis based on the DIAGRAM consortium

Risk factor	I^2	IVW model *		
		OR	95% CI	*p* value
Adulthood BMI	51	1.46	1.35, 1.58	3.30E-20
Visceral fat mass	58	2.18	1.67, 2.85	1.21E-8
Alanine	-	0.62	0.45, 0.87	0.006
Body fat percentage	61	1.71	1.46, 2.02	9.61E-11
Age at menarche	31	0.88	0.83, 0.95	3.00E-4
Insomnia	32	1.17	1.10, 1.25	2.90E-6
Systolic blood pressure	32	1.26	1.11, 1.43	4.80E-4
Lifetime smoking	15	1.19	0.93, 1.51	0.162
Isoleucine	45	1.23	0.91, 1.65	0.176
Childhood BMI	79	1.78	1.26, 2.51	0.001
Caffeine intake	-	1.10	0.92, 1.32	0.283
Birthweight	-	0.72	0.59, 0.88	1.58E-3
Testosterone	-	0.79	0.62, 1.00	0.048
SHBG adjusted for BMI	-	0.61	0.51, 0.72	1.18E-8
Valine	-	1.27	0.92, 1.77	0.148
Leucine	-	1.34	0.90, 1.99	0.148
HDL cholesterol	72	0.77	0.65, 0.91	0.002
Total cholesterol	54	0.94	0.83, 1.07	0.351
Adulthood height	23	0.97	0.94, 1.00	0.039
Alanine aminotransferase	0	1.01	0.99, 1.03	0.315
Coffee consumption	33	1.50	1.13, 1.98	0.005
Phenylalanine	-	1.43	0.95, 2.15	0.083
Tyrosine	-	0.73	0.53, 1.02	0.066
Sodium (urinary)	66	2.75	1.07, 7.09	0.036
IL-6 receptor subunit α	-	0.98	0.95, 1.01	0.148
IL-1 receptor antagonist	-	1.10	0.82, 1.49	0.522
LDL cholesterol	57	0.99	0.88, 1.13	0.957
Giant cell arteritis	0	1.07	1.01, 1.13	0.015
Fetuin-A levels	-	0.97	0.92, 1.03	0.276

CI indicates confidence interval; IVW, inverse-variance weighted; SHBG, sex hormone binding globulin; SNP, single nucleotide polymorphism.

ESM Fig 1. Rationale and key assumptions for Mendelian randomization study

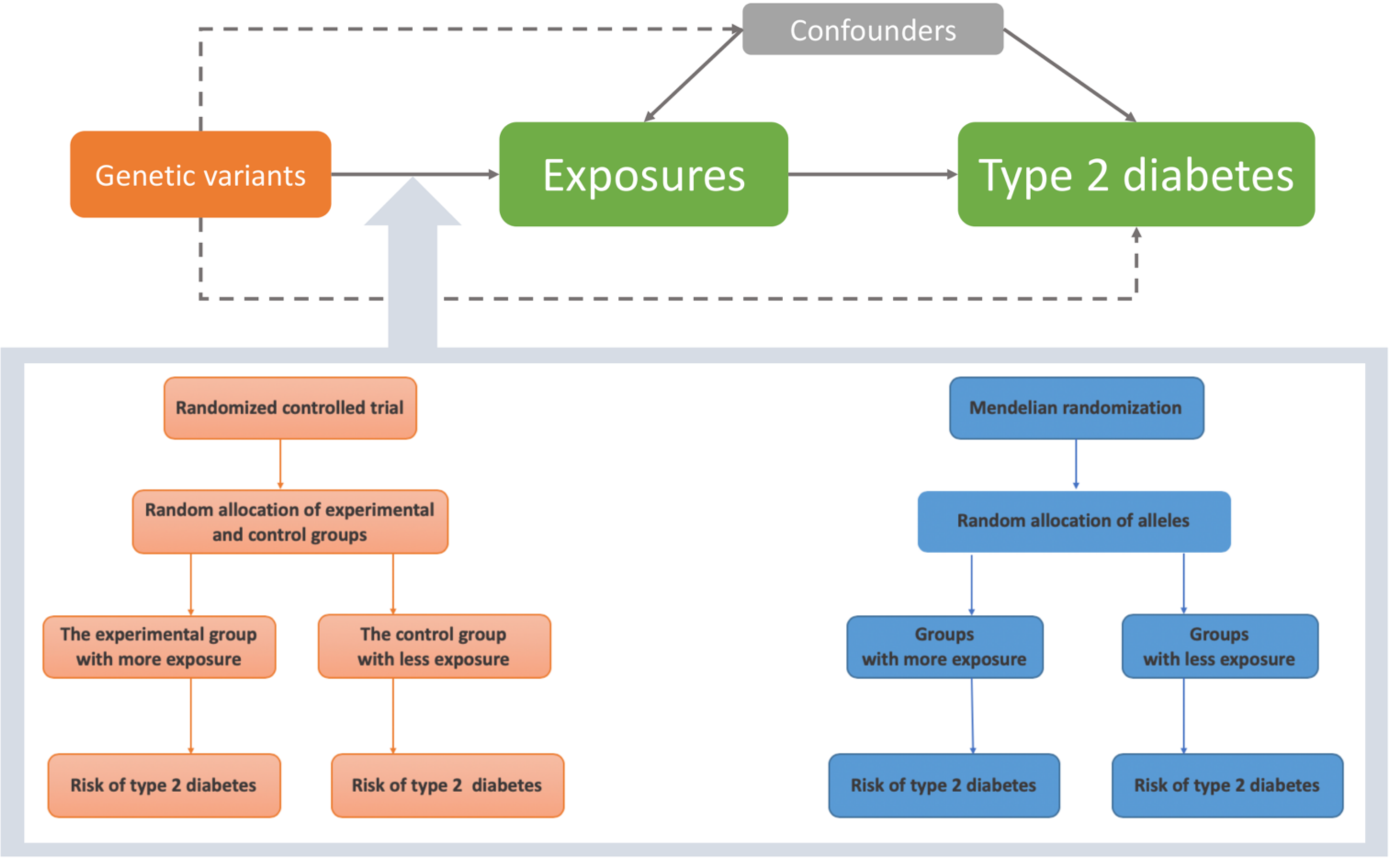

ESM Fig 2. Scatterplot of the alcohol-associated single nucleotide polymorphisms (SNPs) with alcohol consumption (standard deviations of log-transformed drinks/week) and type 2 diabetes (T2D)

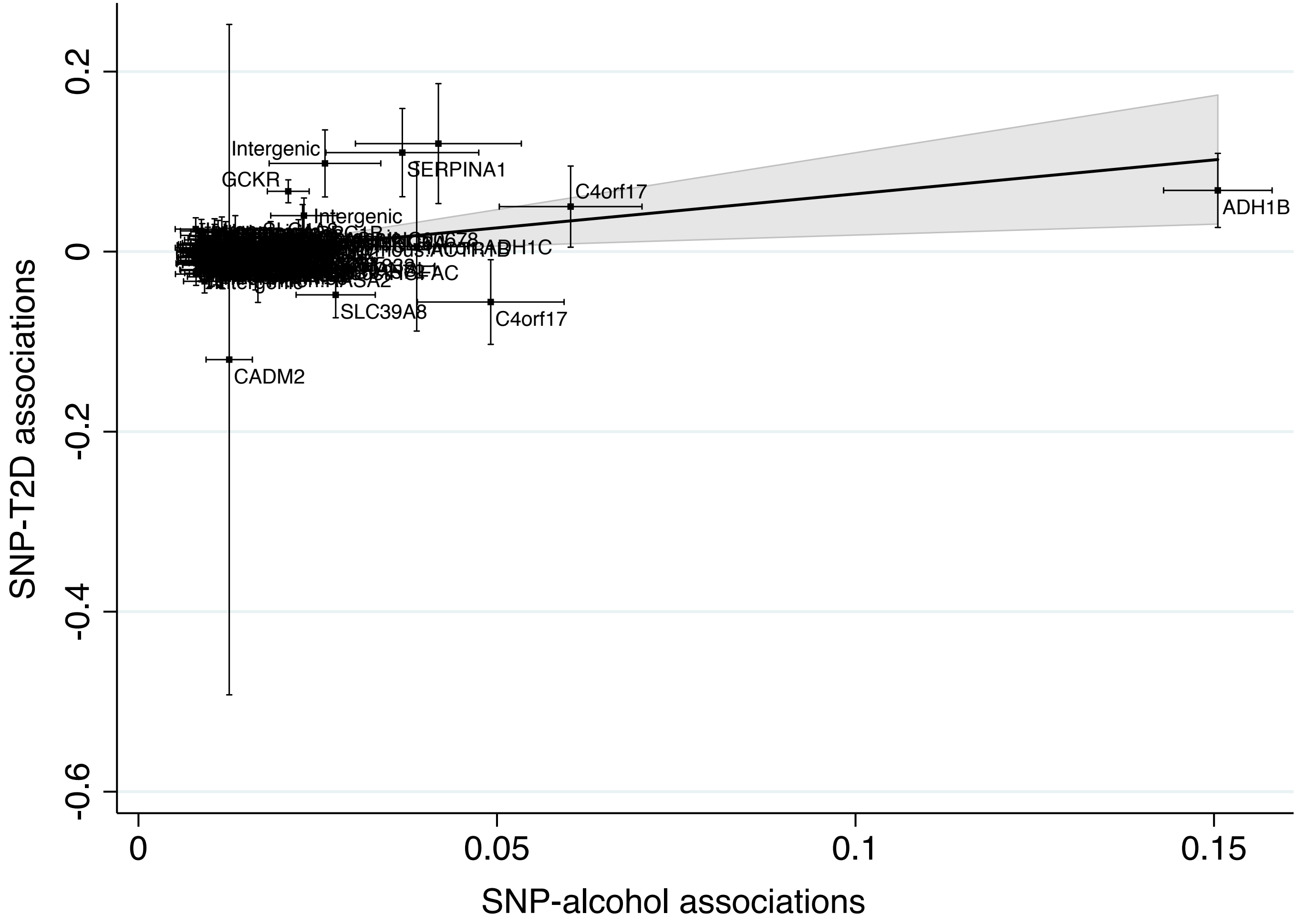

The solid line and shaded area represent the MR-Egger causal estimate with its 95% confidence interval, respectively.
